# Gauging Iron–Sulfur Cubane Reactivity from Covalency: Trends with Oxidation State

**DOI:** 10.1021/jacsau.4c00213

**Published:** 2024-04-05

**Authors:** Liam Grunwald, Daniel F. Abbott, Victor Mougel

**Affiliations:** § Department of Chemistry and Applied Biosciences (D-CHAB), Swiss Federal Institute of Technology Zürich (ETHZ), Vladimir-Prelog-Weg 2, CH-8093 Zürich, Switzerland

**Keywords:** X-ray absorption spectroscopy, iron−sulfur cubanes, oxidation states, bonding, covalency, electron transfer

## Abstract

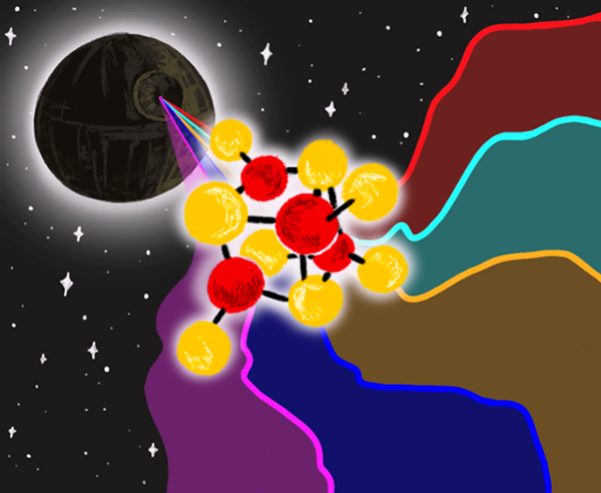

We investigated room-temperature metal and ligand K-edge X-ray absorption (XAS) spectra of a complete redox series of cubane-type iron–sulfur clusters. The Fe K-edge position provides a qualitative but convenient alternative to the traditional spectroscopic descriptors used to identify oxidation states in these systems, which we demonstrate by providing a calibration curve based on two analytic methods. Furthermore, high energy resolution fluorescence detected XAS (HERFD-XAS) at the S K-edge was used to measure Fe–S bond covalencies and record their variation with the average valence of the Fe atoms. While the Fe–S(thiolate) covalency evolves linearly, gaining 11 ± 0.4% per bond and hole, the Fe–S(μ^3^) covalency evolves asystematically, reflecting changes in the magnetic exchange mechanism. A strong discontinuity manifested for superoxidation to the all-ferric state, distinguishing its electronic structure and its potential (bio)chemical role from those of its redox congeners. We highlight the functional implications of these trends for the reactivity of iron–sulfur cubanes.

## Introduction

Iron–sulfur (FeS) cubane clusters are some of the most abundant and versatile enzymatic metallocofactors, but they are challenging to study due to their Fe_4_S_4_ core’s valence-isomerism and high density of states.^1,2^ Both of these factors are, however, considered key to their rich chemistry.^3−5^ Oxidation state assignments thus rely on variable-temperature, variable-field (VT-VF) ^57^Fe Mössbauer or electron paramagnetic resonance (EPR) spectroscopy. The former is particularly powerful because it probes the individual Fe-atoms. However, it also generally requires the experimentally challenging isotopic enrichment of the sample with ^57^Fe.^6,7^ For FeS cluster-containing enzymes, X-ray absorption spectroscopy (XAS)—especially the X-ray absorption near-edge structure (XANES)—thus may constitute a convenient alternative because it does not require isotopic enrichment of the FeS metallocofactor but can still provide a descriptor that allows qualitative oxidation state assignments of a bulk sample. At the same time, the analysis of the extended X-ray absorption fine structure (EXAFS) allows one to investigate the cofactor geometry.^8−11^ Given the structural similarity of canonically ligated FeS cubanes across the wide range of oxidation states,^12^ it is difficult to delineate factors differentiating their reactivity, besides the redox potential. Relatedly, the ^57^Fe Mössbauer isomer shift, δ, or EPR *g*-tensors, commonly used as descriptors, cannot be linked straightforwardly to reactivity. A better descriptor of the reactivity is the covalency of Fe–S bonds, as long as meaningful trends can be uncovered on a series of well-defined, comparable systems.^13^ The variation of covalency could therefore reveal the potentially distinctive chemical roles of Fe_4_S_4_ cofactors depending on their oxidation state. Furthermore, the inverse relationship between the covalency of an Fe–S bond and the Lewis-base character of its S atom set covalency as a good parameter to predict how the basicity and thus the elusive potential protonation sites in [Fe_4_S_4_(^Cys^S)_4_]^*n*−^ cofactors evolve with the oxidation state. In this context, ligand K-edge XAS^14,15^ has proven to be a unique tool to quantitatively measure metal–ligand bond covalencies.^16^

**Scheme 1:**
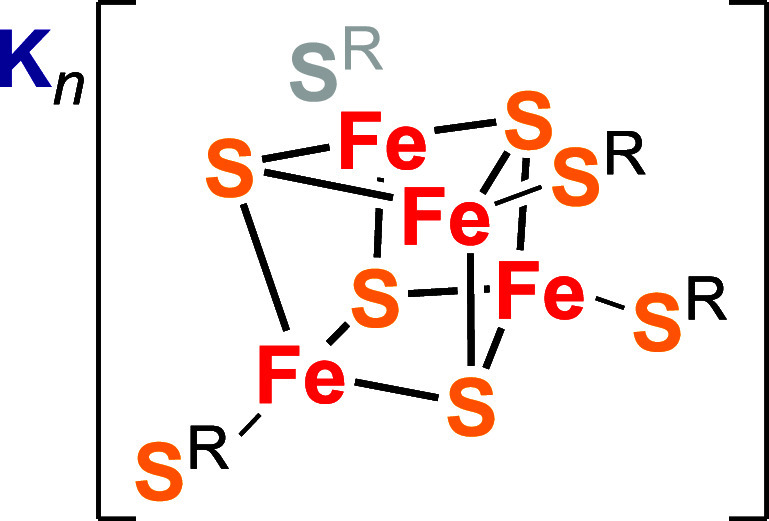
Molecular Structure of the [Fe_4_S_4_(^Cys^S)_4_]^*n–*^ Cofactor Model, K_*n*_[Fe_4_S_4_(DmpS)_4_] (Where *n* = 0-4) R refers to 2,6-dimesitylphenyl (Dmp).^12^

To address these issues, we report here the room-temperature XAS spectroscopic signatures of our recently reported series of biomimetic [Fe_4_S_4_(SR)_4_]^*n*−^ complexes, covering all redox states accessible by cycling the ferrous/ferric (Fe^II^/Fe^III^) states of the individual Fe atoms (Scheme 1).^12^ This allows us to provide a series of benchmark parameters and to propose a calibration curve for qualitative [Fe_4_S_4_]^*n*+^ (*n* = 0–4) oxidation state assignments based on the analysis of Fe K-edge XAS spectra. Using S K-edge high energy resolution fluorescence detected XAS (HERFD-XAS), we estimate the evolution of the Fe–S bond covalency during oxidation state changes of the Fe_4_S_4_ core and relate these to the oxidation state dependent differences in the chemical reactivity of canonical FeS cubanes.

## Results

**Figure 1:**
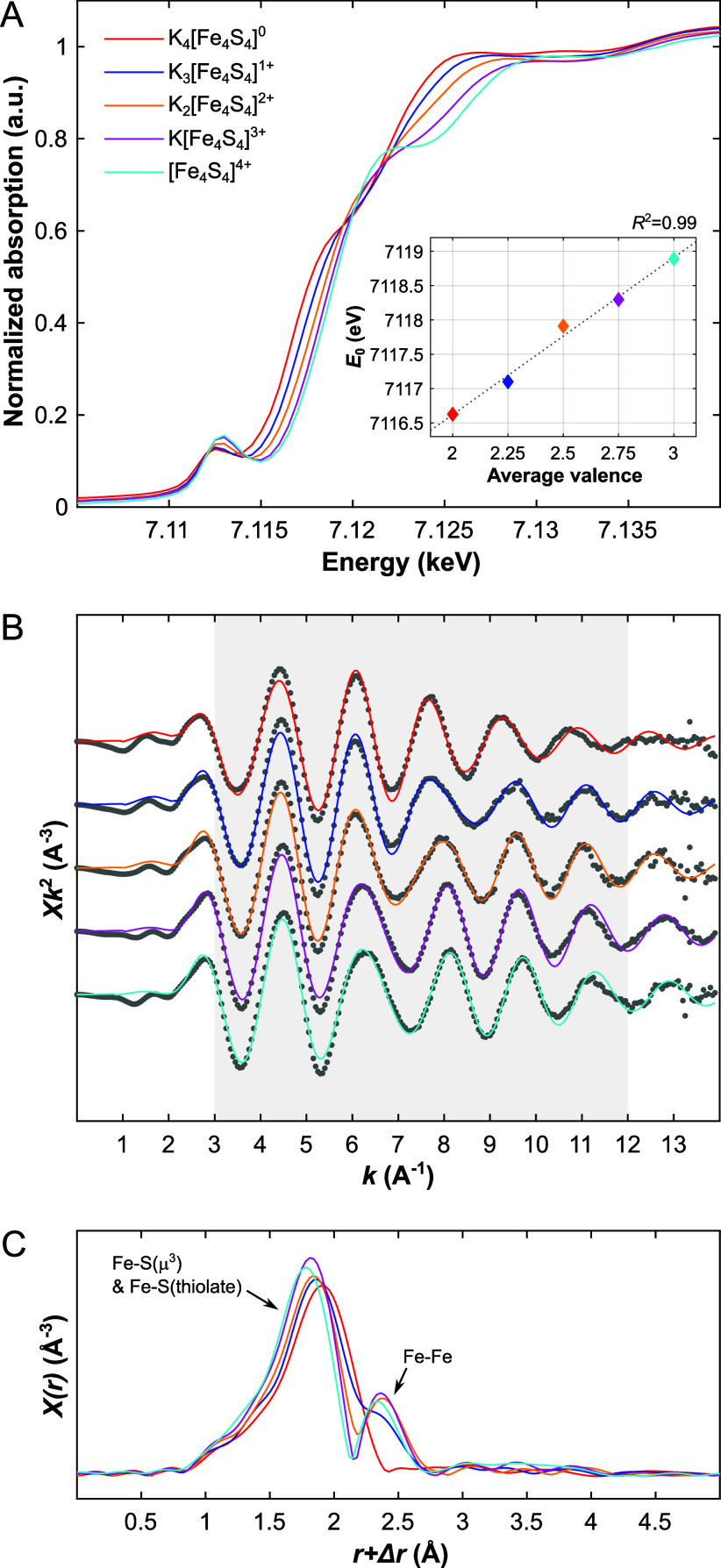
(A) Normalized Fe K-edge XAS spectra of the K_*n*_[Fe_4_S_4_(DmpS)_4_] (*n* = 0–4) powdered samples measured at room temperature. The inset shows the correlation between the average Fe oxidation state (average valence) and the energy of the inflection point (*E*_0_); a linear fit of these data is presented as a *dotted gray line*, indicating an increase in *E*_0_ of 0.57 ± 0.03 eV per 1-electron oxidation. (B) Unfiltered EXAFS spectra of the K_*n*_[Fe_4_S_4_(DmpS)_4_] (*n* = 0–4; *gray dots*) in *k*-space, and the corresponding fits (*colored lines*). The range over which the fit was carried out is shaded in *gray*. (C) FT-EXAFS spectra (*k*^2^-weighted) of K_*n*_[Fe_4_S_4_(DmpS)_4_] (*n* = 0–4).

The Fe K-edge XAS spectra of the FeS cubane redox series, K_*n*_[Fe_4_S_4_(DmpS)_4_], show the typical structure of (pseudo)tetrahedrally coordinated high-spin Fe atoms with a discernible 1*s* → 3*d* pre-edge transition at ca. 7.112 keV and a shoulder at ca. 7.120 keV within the absorption edge (Figure 1A).^17,18^ According to fits to the spectra (Figures S4 and S7), the absorption edge, the pre-edge transition, and the shoulder (mid-edge transition) shift to lower energy and lose intensity when lowering the [Fe_4_S_4_]^*n*+^ oxidation state from *n* = 4 to *n* = 0. This occurs in a nearly linear fashion (Tables S3 and S4 and Figures S6, S8). The intensity decrease of the pre-edge transition can be linked to lesser electric-dipole 3*d*-4*p* mixing, which is governed not only by the oxidation state, but also by the ligand field’s symmetry.^17^ In contrast, the intensity of the white line increases upon reduction of the core. These observations are in line with previous work on three out of the five oxidation states discussed here, which were studied in the nitrogenase iron protein (FeP).^17^ To quantify the shift of the absorption edge energy, *E*_0_, with oxidation state, we evaluated two methods: (*i*) the evolution of the position of the inflection point of the absorption edge, as determined by the highest peak intensity of the first derivative (Figure S5), and (*ii*) the shift of the midpoint of the smoothed step-function used as background in a deconvolution of the spectra (Figure S7). Supporting a robust spectral trend, both methods provided *E*_0_-shift values within the error of another. While method (*i*) suggests a gain of 0.57 ± 0.03 eV per additional hole in the core (Figure 1A, *inset*), method (*ii*) resulted in 0.59 ± 0.08 eV (Figure S9 and Note S3). For method (*i*), which we consider to be the more convenient option, the linear relationship between the average Fe oxidation state (average valence, *V*_Fe_, ranging from 2 to 3) and *E*_0_, is given by eq 1:
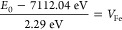
1

The shifts determined from both methods are slightly larger than the average value of 0.45 eV reported for the two redox transformations of the nitrogenase FeP.^17^ Overall, these changes might appear small, but given the calibration curve, either the first derivative or a suitable deconvolution of the Fe K-edge XANES spectrum of an FeS cubane-containing enzyme should provide a quick and qualitative estimate for the majority of the [Fe_4_S_4_]^*n+*^ oxidation states within a possibly heterogeneous sample. The redox reactions are further reflected by significant changes in the Fourier-transformed (FT) EXAFS spectra of the cubanes. Going from [Fe_4_S_4_]^3+^ to [Fe_4_S_4_]^0^, the amplitude of the first-shell peak decreases visibly and the peak broadens, while the second-shell peak diminishes upon reduction of [Fe_4_S_4_]^2+^ to [Fe_4_S_4_]^1+^ and even vanishes completely upon attaining the all-ferrous state ([Fe_4_S_4_]^0^). The merging of the first and second shell peaks into a single, broad peak as [Fe_4_S_4_]^3+^ is reduced to [Fe_4_S_4_]^0^ is further reflected in the bonding distances, which were derived from fitting the EXAFS spectra (Figures 1B and S3) and are reported in Tables S1 and S2. In general, we observe that the first shell (Fe–S) shifts to higher *r*-values while the second shell (Fe–Fe) shifts to lower *r*-values (Figure 1C). This corresponds to a lengthening of the average Fe–S bond length and a simultaneous shortening of the average Fe–Fe bond length, as [Fe_4_S_4_]^3+^ is reduced to [Fe_4_S_4_]^0^. These trends observed in the EXAFS-derived bonding distances reproduce fairly the trends we determined by single crystal X-ray diffraction at 100 K.^12^ Furthermore, as discussed in the case of FeP,^17^ we note that the trends observed in the EXAFS spectra are consistent with the development of a less regular cluster structure upon reduction. This is in line with the disorder of the [Fe_4_S_4_]^0^ core, which we identified crystallographically in K_4_[Fe_4_S_4_(DmpS)_4_]—a structural manifestation of the broken-symmetry *S* = 4 spin ground state.^12^

Beyond the oxidation state and coordination geometry derived from the Fe K-edge, XAS at the S K-edge provides direct insight into the Fe–S bond covalency *via* analysis of the S 1*s* → ψ* pre-edge transition. In a simplified view, ψ* refers to a (weighted) sum over all empty or half-filled antibonding molecular orbitals of the FeS cubane. These S 1*s* → ψ* transitions are formally forbidden. However, they become dipole allowed and gain intensity if the unoccupied Fe 3*d* orbitals are covalently mixed with the S 3*p* orbitals.^19^ The pre-edge intensity is then observed as the pure dipole-allowed S 1*s* → S 3*p* transition, which is weighted by the covalency, α^2^:
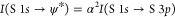
2
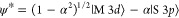
3and α^2^ accordingly expresses the amount of S 3*p* character in the vacant metal orbitals. The net renormalized intensity of the 1*s* → ψ* pre-edge transition is called the dipole strength, *D*_0_. It scales linearly with the Fe–S bond covalency, but it does so differently for a thiolate or a sulfide, due to their distinct transition dipole moment (; 8.05 for S(thiolate) and 6.54 for S(μ^3^), respectively), according to eq 4:^20,21^
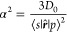
4

Owing to its much better resolved pre-edge fine structure compared to conventional XAS, HERFD-XAS allows for a particularly meaningful discussion of the pre-edge’s attributes, even at room-temperature.^22^ This is especially relevant in spectra of [Fe_4_S_4_(SR)_4_] complexes because the 1*s* → ψ* pre-edge peak is composed of two main lines; one corresponding to the transition of the sulfide S atoms at ca. 2470.1 eV, and one of the thiolate’s S at ca. 2470.9 eV.^23,24^ However, the pre-edge’s intensity and its line shape observed *via* HERFD-XAS do not necessarily correlate with the attributes of the pre-edge observed in conventional transmission or fluorescence detected XAS. In order to evaluate whether the determination of the Fe–S bond covalencies based on the HERFD-XAS data agree with those determined by conventional XAS, we measured a series of standards ([Et_4_N]_2_[Fe_4_S_4_(PhS)_4_], [Et_4_N]_2_[Fe_4_Se_4_(PhS)_4_] and [Et_4_N]_2_[Fe_4_S_4_(PhSe)_4_], Figure S10) by HERFD-XAS. Notably, we obtained covalency values for these compounds that are in very close agreement with those determined by Solomon and co-workers using conventional XAS (see Note S1 and Figure S11),^23−25^ emphasizing that the linear equations established for conventional XAS are applicable to HERFD-XAS within reasonable approximations.

**Figure 2:**
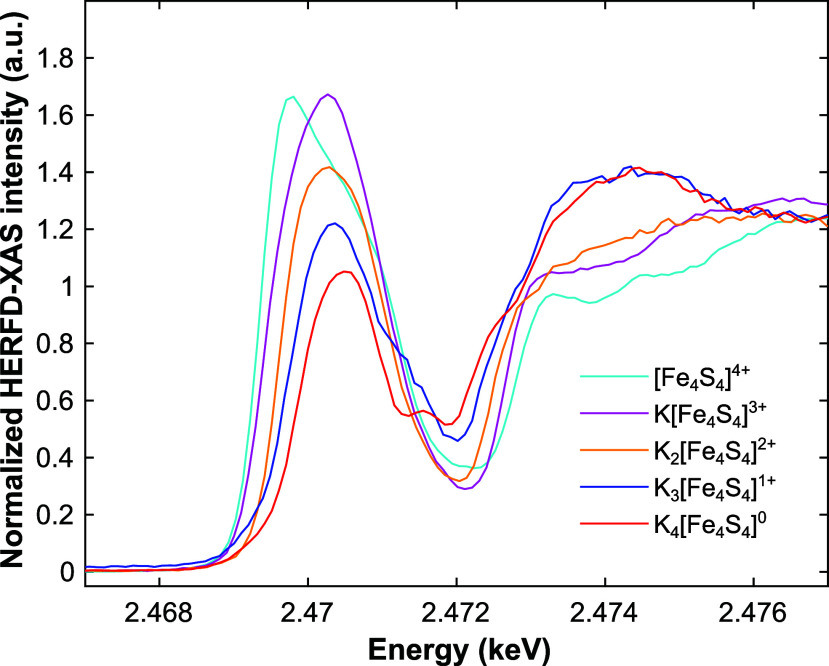
Normalized S K-edge HERFD-XAS spectra of K_*n*_[Fe_4_S_4_(DmpS)_4_] (*n* = 0–4) recorded on powdered samples at room temperature *in vacuo*.

In the S K-edge HERFD-XAS spectra of the FeS cubane redox series, K_*n*_[Fe_4_S_4_(DmpS)_4_] (*n* = 0–4), the pre-edge peak main lines corresponding to both transitions visibly shift to higher energies upon reduction (Figures 2, S12, and S13; Table S5). This is consistent with existing literature and is ascribed to the lower effective nuclear charge of Fe, leading to higher binding energies for the *d*-electrons, and thus to a higher energy for these pre-edge transitions.^17,25^ In turn, this supports the fact that all redox events are (to a large extent) Fe-centered, as invoked by our ^57^Fe Mössbauer studies, the Fe K-edge data presented above, as well as the oxidation state independent S K_α_ and K_β_ X-ray emission spectra (Figures S16 and S17).^12,17^ Interestingly, the transitions of the S(μ^3^) and S(thiolate) ligand contributions are already visually discernible for some of the oxidation states by HERFD-XAS analysis (particularly [Fe_4_S_4_]^0^, [Fe_4_S_4_]^1+^ and [Fe_4_S_4_]^4+^; Figure 2), and can be appropriately fitted in all cases (Figure S12; refer to the Supporting Information for fitting details).

**Figure 3:**
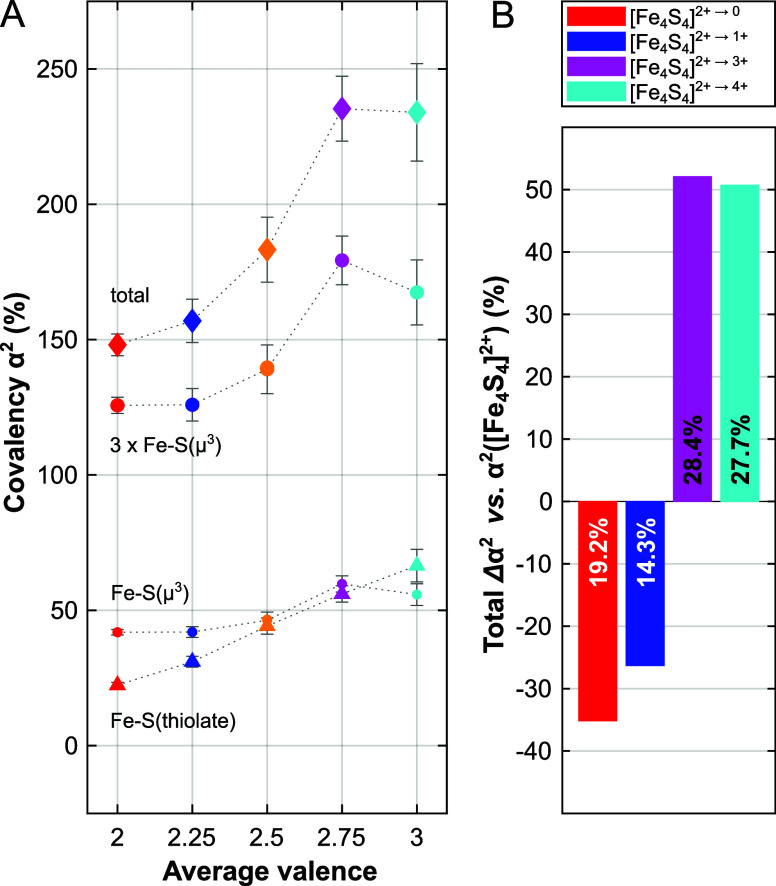
(A) Variation of covalency, α^2^, and its individual contributions with the average valence of the Fe-atom. While the total α^2^ is shown as *colored diamonds* ([Fe_4_S_4_]^4+^: *cyan*, [Fe_4_S_4_]^3+^: *magenta*, [Fe_4_S_4_]^2+^: *yellow*, [Fe_4_S_4_]^1+^: *blue*, [Fe_4_S_4_]^0^: *red*), contributions arising from sulfide- and thiolate-bonding are presented as *dot* and *triangle* shaped markers, respectively. *Dotted gray lines* are intended to guide the eye. Approximate error bars are shown in *gray* color; for details on the error estimation, refer to the Supporting Information. (B) Net change in the covalency upon redox transformations of the [Fe_4_S_4_]^2+^ cubane per Fe-atom: superreduction to [Fe_4_S_4_]^0^ (*red*), ferredoxin (Fd) type reduction to [Fe_4_S_4_]^1+^ (*blue*), high-potential iron–sulfur protein (HiPIP) type oxidation to [Fe_4_S_4_]^3+^ (*magenta*) and superoxidation to [Fe_4_S_4_]^4+^ (*cyan*). The covalency change relative to the resting oxidation state ([Fe_4_S_4_]^2+^ in percent (%) is indicated on the individual bars: *white font* for covalency decrease, *black font* for covalency increase.

The resulting individual Fe–S bond covalencies, extracted according to our fitting routine (Figure 3A), do not display a regular variation across oxidation states: while the total covalency increases with the Fe_4_S_4_ oxidation state from [Fe_4_S_4_]^0^ up to [Fe_4_S_4_]^3+^—more so with every additional hole in the core—it seems to plateau upon superoxidation to [Fe_4_S_4_]^4+^ (Figure 3A). Considering the covalency per Fe-based 3*d*-hole, it even decreases marginally (Table S6). While ^57^Fe Mössbauer, Fe K-edge XAS, and UV–vis electronic absorption spectroscopy did not reveal such discontinuities in the trends among the redox series, the distinction of the [Fe_4_S_4_]^4+^ complex from the other oxidation states is nevertheless in good agreement with our previous structural investigations.^12^ This is corroborated by the room-temperature EXAFS analysis presented here: in good agreement with the corresponding single crystal XRD structures, the Fe–Fe distances increase with oxidation state from [Fe_4_S_4_]^0^ up to [Fe_4_S_4_]^3+^ but shorten upon superoxidation to [Fe_4_S_4_]^4+^ (Tables S1 and S2).

## Discussion

### Electronic Structure Effects during Redox and the [Fe_4_S_4_]^4+^ Complex as an Outlier

Such an asystematic evolution of covalency vs Fe valence hints toward an electronic structure effect at the root of the phenomenon. The Fe–S(thiolate) covalency evolves regularly with oxidation state, and a linear fit indicates a gain of 11 ± 0.4% covalency per Fe–S(thiolate) bond for each additional hole in the core (Figure 3A). Therefore, covalency appears to decrease only for the S(μ^3^) contribution (Figure 3A and Table S5), and the discontinuity seems to be manifested within the [Fe_4_S_4_]^4+^ core itself. In this regard, the magnetic exchange mechanism within the Fe_2_S_2_ subunits contributes to the localization of holes/electrons: superexchange typically results in antiferromagnetic coupling (i.e., lower spin states) and localized states, whereas double-exchange leads to ferromagnetic coupling (i.e., high spin states) and mediates delocalization.^23^ As covalency increases, the magnitude of superexchange increases (quadratically) at the cost of double-exchange.^23^ These trends are effectively reflected in the FeS cubane’s spin ground states: [Fe_4_S_4_]^0^ and [Fe_4_S_4_]^1+^ display low Fe–S covalencies, thus they have low superexchange, and show *S* = 4, and *S*=[3/2, 1/2] spin ground states, respectively, while [Fe_4_S_4_]^3+^ and [Fe_4_S_4_]^4+^, exhibit strong covalency, high superexchange and populate their lowest possible spin ground states (*S* = 1/2 and *S* = 0, respectively).^12^ Ultimately though, the vibronic coupling is the driving force for the valence- and spin topology in canonically ligated clusters.^1,23,26,27^ Therefore, we hope that ^57^Fe nuclear resonance vibrational spectroscopy (^57^Fe NRVS) investigations combined with DFT simulations, both currently underway, will allow us to better elucidate how the vibrational structure evolves with the number of holes in the core.^28^

Overall, the evolution of the S 3*p* character in the Fe 3*d* holes with the Fe valence (Figure 3A) suggests that each oxidation (except the one to the all-ferric state) involves a stronger participation of ligand orbitals in the redox process than the previous one. To relate this to protein function, Figure 3B shows the total covalency changes per Fe atom upon 1-, and two-electron redox in the series studied here, relative to [Fe_4_S_4_]^2+^, the central oxidation state of the redox series and the resting state of Fe_4_S_4_ cofactors in electron transfer enzymes. These values could be extracted from the integrals of the difference spectra, as shown in Figures S14 and S15, or from our fitting routine, producing qualitatively identical results. Evidently, the covalency changes, Δα^2^^2^ upon 1- or 2-electron oxidation (ca. + 28%) exceed the changes upon 1- or 2-electron reduction (−14% and −19%, respectively). The magnitude of any Δα^2^-value relates to the electronic relaxation energy, *E*_rlx_, of the Fe 3*d* valence electrons by causing a change in effective nuclear charge, Δ*q*_rlx_, where^29−31^
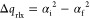
5Here, i and f refer to the initial and final states of redox, respectively. Accordingly, oxidations of [Fe_4_S_4_]^2+^ should result in significantly larger electronic relaxation effects in comparison to its reductions. The functional implications of this difference are discussed below. We attempted to corroborate this trend by complementary X-ray photoelectron spectroscopy (XPS) studies at the Fe 2*p* core-level (refer to the Supporting Information; Figures S18 and S19; Table S7), which was proposed to relate favorably to the trends in the Fe 3*d* valence-level based on investigations of *tetra*(chloride) and *tetra*(thiolate) complexes of Fe^II^.^30^ In the present case, however, the interpretation of the Fe 2*p*_3/2_ spectra remained rather inconclusive. As shown in Figure S19, the Fe 2*p*_3/2_ peak binding energy is not particularly sensitive to the Fe valence state and does not correlate systematically. The binding energy instead remains stable from the [Fe_4_S_4_]^0^ to the [Fe_4_S_4_]^2+^ oxidation state and then increases upon further oxidation to [Fe_4_S_4_]^3+^ and [Fe_4_S_4_]^4+^. Additional fitting and deconvolution of the Fe 2*p* spectra was attempted, but did not yield meaningful results at this time due the high complexity arising from the multiplet splitting and shakeup satellite peak structure that is expected for delocalized high-spin Fe valence states.^32−34^ We do note, however, that the S 2*p* binding energy correlates exceedingly well with oxidation state, scaling linearly and gaining 0.32 ± 0.02 eV per additional hole (Figures S18 and S19; Table S7). This is in strong agreement with the results of the S K-edge HERFD-XAS measurements and indicates that the S 2*p* binding energy may serve as a better indicator of the [Fe_4_S_4_]^*n*+^ core valence state than that of Fe 2*p*—a consequence of the strongly covalent bonding between Fe and S.

### Linking Covalency and Chemical Reactivity

Beyond electron transfers, the measured Fe–S covalencies may constitute a powerful tool to qualitatively predict the reactivity of a FeS cubane in a given oxidation state. Among the redox series, the [Fe_4_S_4_]^2+^ and [Fe_4_S_4_]^3+^ oxidation states show covalencies of the Fe–S(thiolate) and Fe–S(μ^3^) bonds within error of one another (Figure 3A). Upon reduction or superreduction, however, α^2^ of the Fe–S(thiolate) bond drops below the value of α^2^ of Fe–S(μ^3^), while upon superoxidation, α^2^ of the Fe–S(thiolate) bonds exceeds that of Fe–S(μ^3^). In [Fe_4_S_4_]^3+^ and [Fe_4_S_4_]^4+^ the covalency per hole approaches even 50% (Table S6), and the covalency per Fe–S(thiolate) bond exceeds 50% (Figure 3A and Table S5). These high covalency values indicate that the corresponding Fe^2.75^-S(thiolate) bonds found in [Fe_4_S_4_]^3+^ and Fe^III^–S(thiolate) bonds found in [Fe_4_S_4_]^4+^ can be homolytically cleaved rather easily, making the oxidized clusters, and particularly the superoxidized cluster, distinctly available for reductive site-differentiation and/or subsequent cluster interconversion reactions. Evidence for reactivity of this kind was reported by Tatsumi and co-workers, including site-differentiation,^35^ cluster scission,^36^ as well as putative cluster fusion^37^ of amide- and thiolate-ligated [Fe_4_S_4_]^4+^ complexes.^38^ However, the biological relevance of the all-ferric oxidation state as an intermediate is yet to be disclosed.^12,39,40^ Due to their diamagnetism and high reactivity, [Fe_4_S_4_]^4+^ intermediates may be challenging to identify, but the electrochemical potentials reported by our group and that of Tatsumi suggest that they can be generated at a physiologically pertinent electrochemical potential (<+1 V vs NHE).^12,39,41^

For superreduction, the covalency of the [Fe_4_S_4_]^0^ core is similar as in [Fe_4_S_4_]^1+^ (Figure 3A and Table S5), and the ligand character per hole is the same (Table S6). This suggests that the all-ferrous oxidation state can be attained with a marginal electronic restructuring. In turn, the Fe–S(thiolate) covalency decreases significantly (ca. 9% per bond; to 22%). We thus propose that the stability of the all-ferrous oxidation state in an enzymatic system is ultimately controlled by how well the second sphere mitigates Coulombic repulsion between the overall neutral, but electron rich [Fe_4_S_4_]^0^ core and its poorly covalent anionic cysteine ligands.^42^ Effective electrostatic stabilization is e.g. provided by the nitrogenase FeP, whose cofactor is solvent-exposed and surrounded by four α-helix dipoles.^43−46^ If this stabilization is not provided, all-ferrous cubanes suffer from ligand loss, and may form aggregates or interact with oxidizing substrates.^42^ Relatedly, the low covalencies of the Fe–S(μ^3^), but particularly of the Fe–S(thiolate) bonds in the [Fe_4_S_4_]^0/1+^ clusters render the S-sites significantly more basic than the sites in their oxidized congeners. We thus suspect that these reduced clusters are more prone to be involved in proton-transfer (PT), or even concerted proton–electron transfer (CPET) reactivity compared to their oxidized analogues. The low covalency of the Fe–S(thiolate) bonds in [Fe_4_S_4_]^0/1+^ further leads us to support the fact that the likely site of protonation should be the thiolate (^Cys^S), as suspected based on recent spectroscopic and theoretical studies of hydrogenase by Stripp and co-workers.^47,48^

**Figure 4:**
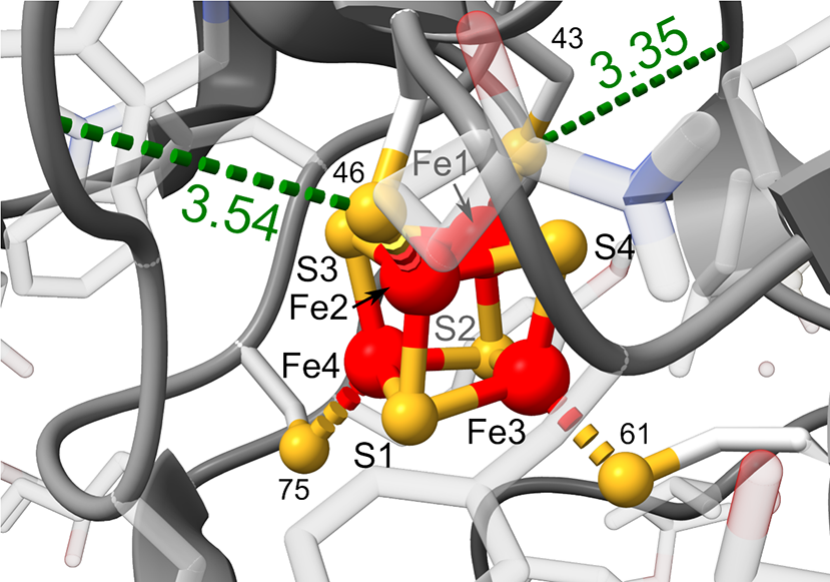
H-bonds (*dashed green*; values in Å; given as distance from the H-bond donor to the acceptor atom) detected in the second sphere of the FeS cofactor in the crystal structure of *thermochromatium tepidum* HiPIP (PDB code 5WQR(49)) in the reduced ([Fe_4_S_4_]^2+^) oxidation state. H-bonds were calculated according to the criteria described by Mills and Dean,^50^ relaxing the angle and distance tolerances by 20° and 0.4 Å, respectively.^51^ This figure was generated using the ChimeraX program suite.^52,53^

Last, Δα^2^ has been proposed to be relatable to the nature of the redox active molecular orbital (RAMO):^25^ the determined covalency values (Figure 3) suggest that the RAMO operative for the [Fe_4_S_4_]^2+/3+^ redox couple found in HiPIP has a stronger localization on the ligands compared to the RAMO of the [Fe_4_S_4_]^1+/2+^ redox couple found in Fd. This is in good agreement with the fact that the RAMO’s ligand character couples the cluster into the HiPIP’s superexchange pathways for electron transfer (ET), which is, in turn, important because—in contrast to the Fd’s surface-exposed cofactors—HiPIP’s cofactors are buried in hydrophobic cavities.^19,25,31,54,55^ Reviewing most recent literature on HiPIP’s structure, this coupling could occur *via* H-bonds from the backbone to the strongly covalently mixed thiolate ligands. Notably, i.e., the residues ^Cys^46 and ^Cys^43 of *thermochromatium tepidum* could fulfill this function (Figure 4).^49^ Intriguingly, the Fe_2_S_2_(^Cys^S)_2_ subcluster possessing the H-bonds (Fe1, Fe2, S3, and S4) is precisely the charge-bearing redox active subcluster, which was suggested based on scXRD,^55^ VT-^1^H NMR,^56,57^ and, more recently, a charge-density analysis at 0.48 Å resolution.^58^

## Conclusions

In summary, we provided for the first time the room-temperature XAS signatures of Fe_4_S_4_ complexes covering all oxidations states within the Fe^II/III^ redox couple of the individual iron atoms at the metal and ligand K-edges. The regular variation of the Fe K-edge energy throughout the complete Fe_4_S_4_ redox series and the provided calibration curve will support the development of a more routine application of Fe K-edge XAS in bioinorganic chemistry, allowing for qualitative but convenient assignments of [Fe_4_S_4_]^*n*+^ oxidation states. Beyond this aspect, we highlight that the evolution of the Fe–S covalency with varying oxidation state can be used to distinguish the reactivities of [Fe_4_S_4_(SR)_4_]^*n*−^ complexes based on the number of electrons/holes in their core. These trends foster our understanding of the functional relevance of elusive electronic structure effects in these systems. In particular, the discontinuity of covalency observed for the all-ferric oxidation state ([Fe_4_S_4_]^4+^) hints toward its possibly distinctive role among its reduced congeners, which we propose to be beyond electron/proton transfer. To support this, we are currently pursuing synthetic, but also further combined theoretical/spectroscopic work. Alternatively, it could also prove to be a useful argument in explaining why [Fe_4_S_4_]^4+^ intermediates are, to date, not yet found/accessed in biological systems.

## Supporting Information Available

Sample preparation procedures as well as details on the experimental setup and instrumentation for the XAS and XPS measurements; additional graphs illustrating all relevant fits as well as details on the performed data analysis routines (PDF)
