# Supplementary material for: Gauging Iron–Sulfur Cubane Reactivity from Covalency: Trends with Oxidation State
Source: JACS Au. 2024 Apr 5;4(4):1315–22. doi: 10.1021/jacsau.4c00213 (PMC11040707; doi:10.1021/jacsau.4c00213)
Supplement: Supplementary file 1 — au4c00213_si_001.pdf [file au4c00213_si_001.pdf]

Supporting Information File for

Gauging Iron-Sulfur Cubane Reactivity from Covalency: Trends with Oxidation State

Liam Grunwald, Daniel F. Abbott and Victor Mougel*

Department of Chemistry and Applied Biosciences (D-CHAB), Swiss Federal Institute of Technology Zürich (ETHZ), Vladimir-Prelog-Weg 2, CH-8093 Zürich, Switzerland.

Sample Preparation, Measurement and Data Analysis ... **2**

Fe K-edge XAS data ... *2*

S K-edge HERFD-XAS data ... *2*

XPS data ... *5*

Supporting Figures and Tables ... **6**

References ... **24**

Sample Preparation, Measurement and Data Analysis

All iron-sulfur cubane compounds studied in this work were synthesized according to the procedures published previously by our group elsewhere.[1-2]

Fe K-edge XAS data

X-ray absorption spectra were collected at the BM23 beamline of the European Synchrotron Radiation Facility (ESRF) (Grenoble, France).[3] Measurements were performed using a double-crystal Si(111) monochromator moving in a continuous mode and a pair of flat Si mirrors positioned at 3.0 mrad for harmonic rejection. Spectra were recorded in transmission mode at the Fe K-edge and calibrated using a Fe reference foil at 7112 eV. The ionization chambers were filled a mixture of N_2 and He at 0.89 bar (I_0) and N_2 at 2.2 bar (I_1 and I_2). Finely ground sample powders were pressed into 1 mm thick pellets using boronnitride as a diluent. The sample mass in each pellet was optimized to give an edge step of roughly 1 absorption unit. All pellets were prepared in a glovebox under inert atmosphere (Ar), which were then sealed individually inside air-tight bags. All samples were protected from exposure to air and moisture by transporting them to the beamline under inert atmosphere (Ar) and by measuring them while still sealed inside their respective air-tight sample bags. Multiple scans were collected for each sample in order to ensure that the final data did not contain any radiation-induced spectral distortions. Because we could not observe any significant difference between three consecutive scans on the same sample position, we were confident that beam-damage effects are small.

X-ray absorbtion near-edge structure (XANES) and extended fine structure (EXAFS) data were analyzed using the Demeter software package.[4] Initial data processing included background subtraction, normalization, and calibration *via* the simultaneously measured metallic Fe reference foil. In all cases, the edge position, E_0, was determined from the primary peak of the first derivative—also referred to as the “inflection point”. Fitting of the absorption edge was performed using MATLAB. The edge jump was fitted using a pseudo-Voigt function (Figure S4) or a smoothed step function (Figure S7), and the pre-edge as well as the mid-edge absorption features were fitted using pure gaussian lines.

Measured spectra were converted to *k*-space, *i.e.* the photoelectron wave vector, and the resulting $\chi(k)$ functions were k^3-weighted and Fourier transformed over 3-12 Å^{-1}. Fitting of the EXAFS data was performed using a theoretical model generated from the corresponding scXRD crystallographic data.[1] EXAFS fitting was performed over a *k*-range of 3-12 Å^{-1} and an *r*-range of 1-2.9 Å. A total of 5 free parameters were used for each fit (ΔE_0, Δr_S, σ^2_S, Δr_{Fe}, and σ^2_{Fe}) to account for the energy shift (ΔE_0) and for changes in the bond lengths (Δr_S and Δr_{Fe}) and the Debye-Waller factors (σ^2_S and σ^2_{Fe}) of the first (Fe-S) and second (Fe-Fe) coordination shells, respectively. The same Δr_S and σ^2_S guess parameters were used to describe all Fe-S scattering paths. Similarly, the same Δr_{Fe} and σ^2_{Fe} guess parameters were used to describe both Fe-Fe scattering paths. Coordination numbers (N) were set to the known values as determined by single-crystal X-ray diffraction. The amplitude reduction factor (S_0^2) was determined to be 0.71 by the fitting the Fe metal reference foil. The results of our EXAFS analysis are summarized in Figure S3, Table S1 and Table S2.

S K-edge HERFD-XAS data

X-ray absorption spectroscopy (XAS) and X-ray emission spectroscopy (XES) measurements were performed at the S K-edge (2.472 keV) at the ID26 beamline of the European Synchrotron Radiation Facility (ESRF) (Grenoble, France).[5] The first harmonic of the undulator source was used, and higher harmonics were rejected using Si-coated mirrors. The incident beam was monochromatized using a cryogenically cooled Si(111) double-crystal monochromator and Si-coated focusing mirrors were used to achieve a spot size of 200 x 100 μm² at the sample position. The dispersive optics of the spectrometer and the sample analysis chamber are always maintained under vacuum.[6] High energy resolution fluorescence detected (HERFD) XAS were measured at the maximum of the K_α emission mainline. The measurements utilized a crystal array spectrometer with six cylindrically bent $LiNbO_3$ (10-4) crystals with a 1 m radius of curvature. For the XAS measurements, the incident energy wasn't specifically calibrated since there was no suitable reference foil at low energies. The calibration was instead based on the previous calibration done at Mn K-edge (6.539 keV). The measurements were performed using a multi-wire gas detector.[6]

XES measurements were performed at both the K_α and K_β emission lines.

All samples were finely ground into powders using a mortar and pestle, then loaded into a custom sample plate (Figure S1, *left*) and compacted by carefully pressing them with a metal spatula. In this configuration, once the sample plate was closed, the sample powders were protected from the atmosphere by a thin, sulfur-free Kapton

foil, pressed between two metal parts of the sample plate (Figure S1, *right*). The sample plates, as well as the Kapton foil were generously provided by the beamline staff.

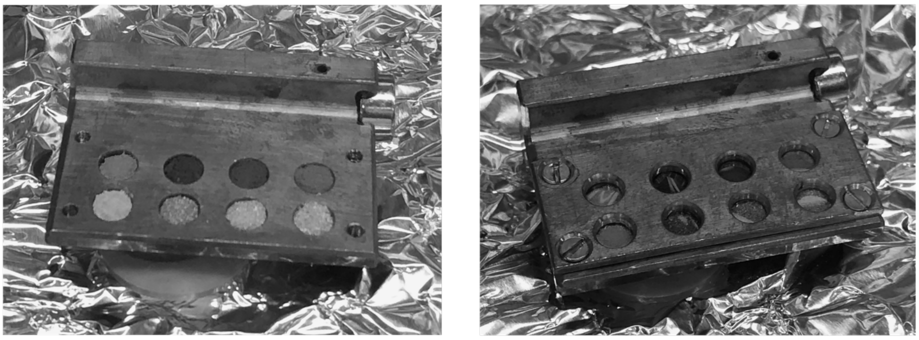

Figure S1. Pictures of the open (*left*) and closed (*right*) custom sample plate filled with sample powders. Note that the loaded samples shown here do not correspond to the investigated FeS cubanes, but were loaded in the same manner. The dimensions of the wells were 5 mm diameter x 1.5 mm depth.

All of the sample preparation and loading was performed inside an Ar-filled glovebox and the sample holders were individually sealed airtight in multiple layers of plastic, allowing inert transport to the beamline. The sample holders were transferred into the vacuum loading chamber of the spectrometer quickly. To assess beam-damage effects, quick near-edge XAS spectra were consecutively recorded on the same position in the sample until differences in the consecutive spectra were noticed. During a 10 s exposure of the sample, we were unable to detect beam-damage induced spectral distortions and, therefore, 10 s scans were performed in 100-150 (HERFD-XAS), 30 (K_α-XES) or 50 (K_β-XES) individual positions throughout the sample, respectively, and their average was used to construct the final spectrum. For some samples, a small number of individual spectra were manually excluded as outliers, because of the high signal-to-noise ratio; This was likely due to minor sample inhomogeneities (*i.e.* crystallite/particle sizes). Furthermore, because self-absorption is a known problem for S K-edge XAS, we tested the dilution of our samples with boron-nitride, as it is commonly done. However, we were unable to observe significant qualitative differences in the spectral attributes between the diluted and undiluted samples, besides an improved signal-to-noise ratio of the undiluted powders. Therefore, in this work, we will present the data recorded on the undiluted samples.
HERFD-XAS data were initially analyzed using the PyMCA software package.[7] This included background subtraction, normalization, and energy-calibration *vs.* the literature-known compounds, $[Et_4N]_2[Fe_4S_4(PhS)_4]$, $[Et_4N]_2[Fe_4S_4(PhSe)_4]$ and $[Et_4N]_2[Fe_4Se_4(PhS)_4]$.[8] The latter were also used in order to compare the covalencies obtained from HERFD-XAS analysis to those obtained from conventional XAS experiments reported in the literature (refer to Note S1 for additional details on this matter).[9-11] The normalized data was then fed into a custom MATLAB routine for fitting of the pre-edge peaks, performing the following operations, which we adapted from the procedures of Solomon and co-workers to the best of our ability:[9-11]

- The rising edge of the absorption spectrum until 2743 eV was fitted with a pseudo-Voigt line.
- The pre-edge transition contribution of the sulfide ligands in $[Et_4N]_2[Fe_4S_4(PhSe)_4]$ was fitted using a Gaussian line of free width and variable position (the fit did not improve when using a pseudo-Voigt line). We obtained a dipole strength of 2.87, which corresponds to a covalency of 43±2% per Fe-S(μ^3) bond (*vide infra*), in reasonable agreement with existing literature (39±3-41±2%; for literature-known synthetic $[Fe_4S_4]^{2+}$ clusters with varying ligand sets).[9-11]
- The pre-edge transition of the thiolate ligands in $[Et_4N]_2[Fe_4Se_4(PhS)_4]$ was fitted using a Gaussian line of free width and variable position (the fit did not improve when using a pseudo-Voigt line). We obtained a dipole strength of 0.97, which corresponds to a covalency of 36% per Fe-S(thiolate) bond (*vide infra*), likewise within error of the existing literature data (35±2%).[9-11] For all subsequent fitting routines, the ratio (but not the absolute peak widths) between the peak widths of the sulfide and thiolate contributions was fixed as determined in the fitting of these two reference compounds, $[Et_4N]_2[Fe_4Se_4(PhS)_4]$ and $[Et_4N]_2[Fe_4S_4(PhSe)_4]$.
- A fit to the data measured on $K_n[Fe_4S_4(DmpS)_4]$ (n=2) was performed using the two peak shapes and positions as determined on the reference compounds containing only thiolate or only sulfide, respectively, with the peak energy positions constrained tightly around the previously determined value (±0.1 eV). Fits to the remainder of the data (n=0,1,3,4) were then generated by manually increasing the

center of the energy position as evident from the structure of the spectra, as well as existing literature,[10] and constraining said position (±0.05 eV). All widths (FWHM) of sulfides refined to values between 1.0 and 1.2 eV, while all thiolate contributions were fairly fitted with widths of 0.8-1.0 eV.

- In this procedure, we suppose that the largest error in covalency arises due to overfitting of the peak positions and widths. Because we constrain the ratio of the two peak widths, errors are more dramatic due to wrong peak positions, influencing, in turn, once again the optimized widths. Therefore, we roughly estimated the error by perturbing the peak positions by ±0.05 eV, and keeping track of the effect on the correspondingly fitted intensities (see uncertainties given in Table S5).
- The intensities of the peak contributions were converted into dipole strength by "renormalization". This means the observed intensity is multiplied by the number of absorber atoms in the molecule (for our complexes this is 8 S atoms) and divided by the number of atoms associated with the transition (4 thiolate S atoms, or 4 sulfide S atoms respectively). Thus, for the Fe_4S_4 complexes studied in this work, the renormalization factor amounted to 2 in all cases.[12-13] Additionally, the dipole strength was calculated per bond, which means that for the $S(\mu^3)$ transition, D_0 was further divided by 3.
- Lastly, the thus obtained dipole strengths (per bond), D_0, were converted to covalency values, α^2, according to the linear equation reported and utilized by Solomon and co-workers (*vide infra*), using a value of 6.54 for the sulfide's transition dipole moment (the denominator of the equation), and 8.05 for the thiolate's:[10, 12-13]

$$\alpha^2 = \frac{3D_0}{\langle s|\hat{\boldsymbol{r}}|p\rangle^2}$$

Note S1. It is known that the HERFD-XAS pre-edge intensity and the pre-edge transition line shape do not correlate directly with the intensity and line shape of the pre-edge transitions in conventional XAS. Hereby, the fact whether the relative intensity of thiolate *vs.* sulfide is maintained in HERFD-XAS compared to conventional XAS is of particular concern, because they could have slightly different resonant emission energies in the RIXS map. Thus, in order to estimate whether the linear equations developed to determine the metal-ligand bond covalency from conventional XAS provide a valid approximation for covalency values determined from the intensity of HERFD-XAS pre-edge intensities, we investigated the S K-edge HERFD-XAS spectra of the well-known compounds, $[Et_4N]_2[Fe_4S_4(PhS)_4]$, $[Et_4N]_2[Fe_4S_4(PhSe)_4]$ and $[Et_4N]_2[Fe_4Se_4(PhS)_4]$ (Figure S10).[8]
As detailed in the description of our data analysis routine above, we did not observe a significant difference between the covalency values determined on these standard compounds by us using HERFD-XAS and the same (and similar) compounds that have been investigated by Solomon and co-workers in the literature (refer to Figures S10 and S11 for the spectra and fitting of the sulfide and thiolate contributions, respectively).[9-11]
Based on this, we therefore believe that—within reasonable approximation—the equations developed to determine covalency from conventional XAS can be used for HERFD-XAS as well.
Furthermore, we wish to highlight that because this work focusses on the comparison of relative covalency changes within a systematic series of compounds, and does not discuss absolute covalency values, the potential systematic error, which is likely on the order of magnitude of a few percent at best, is meaningless in the context of our discussions.

Note S2. An apparent shortcoming of this fitting routine is our methodology of refining the peak width. However, the spectral features, particularly *i.e.* the line shape of the spectrum of the $[Fe_4S_4]^{4+}$ complex suggests that one Gaussian peak for each of the two transitions of the pre-edge peak is not enough. This is indeed known for these kinds of systems, and originates from the configuration interaction (CI).[14] In fact, all systems containing Fe^{II}/Fe^{III} in weak ligand fields are prone to this effect. Fe_4S_4 complexes in particular are notorious for their high density of low-lying excited states and therefore it does indeed make sense that these types of features are relevant, especially in room-temperature HERFD-XAS. However, because the rest of the spectra (for $[Fe_4S_4]^{0/1+/2+/3+}$) could be reproduced fairly well with only two Gaussians (Figure S12), instead of risking an overfitting of the $[Fe_4S_4]^{4+}$ spectrum by a potentially complicated combination of lines, we preferred to stay systematic in our approach and use the same methodology and parameter space as for the other complexes. As evident in Figure S12, the fit of the $[Fe_4S_4]^{4+}$ spectrum is poorer compared to the other complexes. This causes a higher uncertainty in the determined covalency, which perhaps even exceeds the reported value.
Because we obtained the qualitatively identical trend of the total covalencies (Figure 3B) by approximating covalency changes from the simple integrals of the difference spectra (Figure S14 and S15), we are nonetheless confident in the conclusions of our study regarding the special nature of the all-ferric oxidation state of the iron-sulfur cubane.

XPS data

X-ray photoelectron spectroscopy (XPS) measurements were performed on a Sigma II instrument (Thermo Electron) equipped with an Alpha 110 hemispherical analyser. The instrument was operated in large area XPS mode using an Al K_{α} X-ray source at 200 W. All samples were prepared in an Ar-filled glovebox by loading the sample powder into a home-made sample holder that allows for the samples to be transferred into the FEAL chamber under vacuum without being exposed to the ambient atmosphere. The pressure in the XPS analysis chamber was maintained under 5.0 x 10^{-8} mbar during all measurements. Survey scan spectra were collected up to a binding energy of 1100 eV using a pass energy of 50 eV, a step size of 1 eV, and a dwell time of 50 ms. Narrow region scans were collected using a pass energy of 25 eV, a step size of 0.1 eV, and dwell time of 50 ms. All spectra were calibrated to the C 1*s* peak of the thiolate ligand at 284.8 eV (corresponding to the energy expected for C=C-H bonded carbons).
Fitting was carried out in the CasaXPS[15] program package.

Supporting Figures and Tables

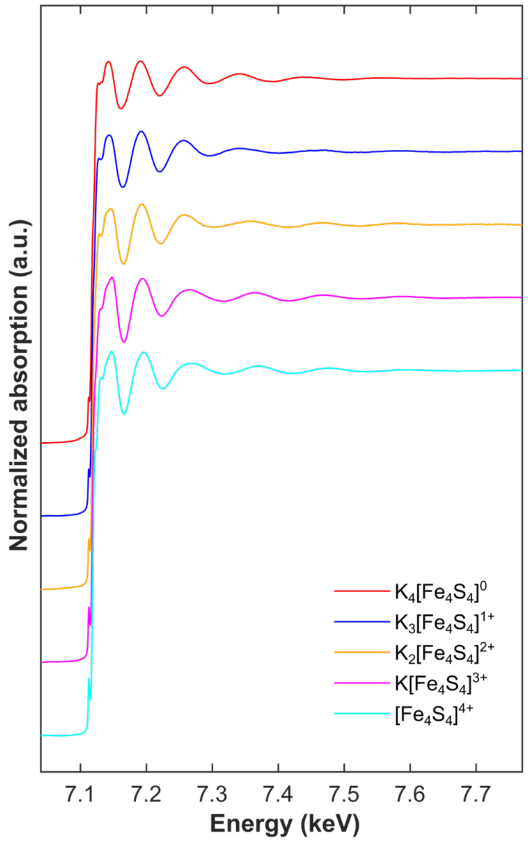

Figure S2. Normalized, unfiltered X-ray absorption spectra of the $K_n[Fe_4S_4(DmpS)_4]$ (n=0-4) powder samples recorded at the Fe K-edge at room temperature: *red*, $K_4[Fe_4S_4]^0$; *blue*, $K_3[Fe_4S_4]^{1+}$; *yellow*, $K_2[Fe_4S_4]^{2+}$; *magenta*, $K[Fe_4S_4]^{3+}$ and *cyan*, $[Fe_4S_4]^{4+}$.

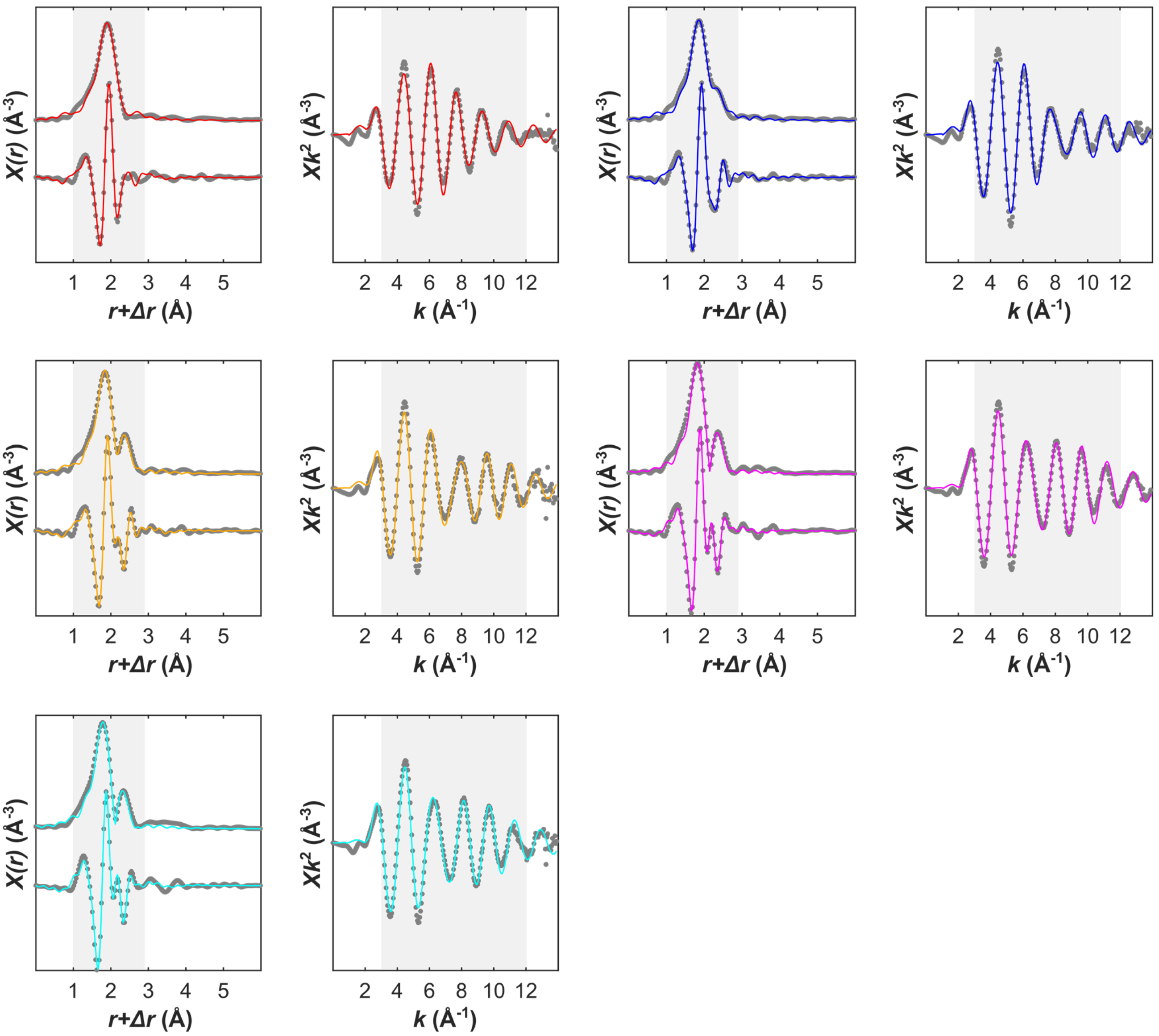

Figure S3. Fitted EXAFS spectra of the $K_n[Fe_4S_4(DmpS)_4]$ (*n*=0-4) powder samples recorded at the Fe K-edge at room temperature: *red*, $K_4[Fe_4S_4]^0$; *blue*, $K_3[Fe_4S_4]^{1+}$; *yellow*, $K_2[Fe_4S_4]^{2+}$; *magenta*, $K[Fe_4S_4]^{3+}$ and *cyan*, $[Fe_4S_4]^{4+}$. The fitted spectra are shown in *r*-space and *k*-space. All Fe χ(k) functions were Fourier transformed over a *k*-range of 3.0-12.0 $Å^{-1}$ and the fitting was performed over an *r*-range of 1.0–2.9 Å, as is indicated by the shaded regions. Data are shown by *dark grey dots* and fits are *coloured lines*.

Table S1. Summary of the EXAFS best fit parameters for the $K_n[Fe_4S_4(DmpS)_4]$ (n=0-4) powder samples recorded at the Fe K-edge at room temperature and *in vacuo*. All Fe χ(k) functions were Fourier transformed over a *k*-range of 3.0-12.0 Å^{-1} and the fitting was performed over an *r*-range of 1.0-2.9 Å. *N* is the coordination number of the scattering path, *d* indicates the refined path length, σ^2 is the Debye-Waller factor, ΔE_0 denotes the energy shift, and the R−factor represents the relative fit error. Coordination numbers were set to the known values determined from single-crystal X-ray diffraction. An S_0^2 value of 0.71 was used for all samples, as was determined by the fitting the Fe metal reference foil.

complex	scattering path	*N*	*d* (Å)	σ^2 (Å^2)	ΔE_0 (eV)	R-factor
$K_4[Fe_4S_4]^0$	S004.1	1	2.269±0.010	0.0031±0.0007	3.74±1.11	0.0212
	S004.2	2	2.315±0.010	0.0031±0.0007		
	S004.3	1	2.383±0.010	0.0031±0.0007		
	Fe01.1	1	2.591±0.021	0.0122±0.0028		
	Fe02.2	2	2.664±0.021	0.0122±0.0028		
$K_3[Fe_4S_4]^{1+}$	S004.1	1	2.245±0.009	0.0027±0.0007	3.88±0.89	0.0187
	S004.2	2	2.291±0.009	0.0027±0.0007		
	S004.3	1	2.359±0.009	0.0027±0.0007		
	Fe01.1	1	2.644±0.015	0.0080±0.0017		
	Fe02.2	2	2.717±0.015	0.0080±0.0017		
$K_2[Fe_4S_4]^{2+}$	S00A.1	1	2.216±0.007	0.0019±0.0006	3.89±0.75	0.0182
	S00B.1	1	2.271±0.007	0.0019±0.0006		
	S007.1	2	2.314±0.007	0.0019±0.0006		
	Fe02.1	1	2.720±0.016	0.0091±0.0016		
	Fe03.1	2	2.751±0.016	0.0091±0.0016		
$K[Fe_4S_4]^{3+}$	S00D.1	2	2.238±0.008	0.0021±0.0006	4.00±0.91	0.0259
	S00B.1	1	2.258±0.008	0.0021±0.0006		
	S00A.1	1	2.309±0.008	0.0021±0.0006		
	Fe03.1	2	2.734±0.021	0.0098±0.0026		
	Fe02.1	1	2.799±0.021	0.0098±0.0026		
$[Fe_4S_4]^{4+}$	S008.1	2	2.216±0.007	0.0029±0.0005	2.58±0.77	0.0186
	S006.1	2	2.263±0.007	0.0029±0.0005		
	Fe02.1	2	2.722±0.018	0.0111±0.0022		
	Fe04.1	1	2.748±0.018	0.0111±0.0022		

Table S2. Average bond lengths extracted from the EXAFS analysis (Figure S3 and Table S1).

complex	Fe-Fe av. (Å)	Fe-S(R) av. (Å)
$[Fe_4S_4]^{4+}$	2.731	2.240
$K[Fe_4S_4]^{3+}$	2.756	2.261
$K_2[Fe_4S_4]^{2+}$	2.741	2.279
$K_3[Fe_4S_4]^{1+}$	2.693	2.297
$K_4[Fe_4S_4]^0$	2.640	2.321

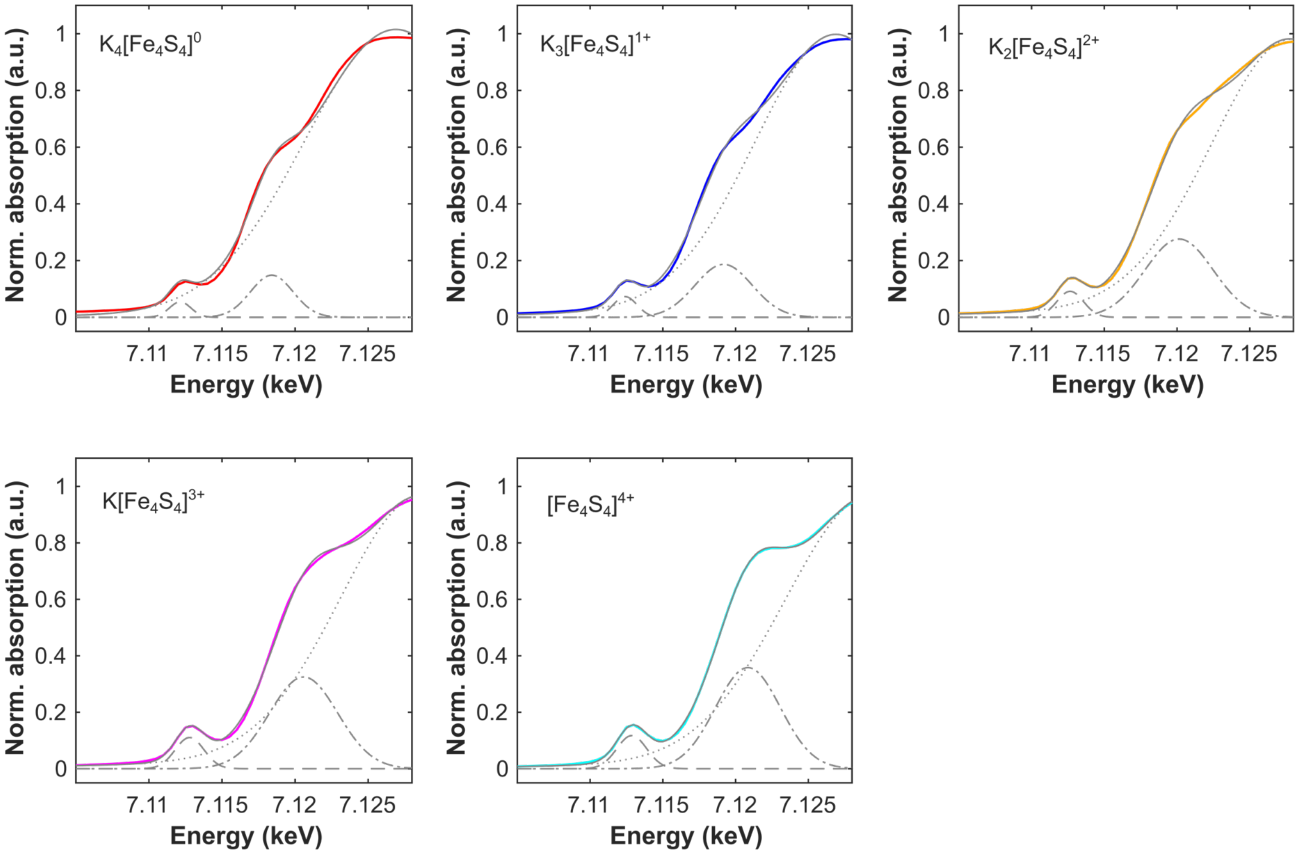

Figure S4. Normalized and fitted Fe K-edge XAS spectra of $K_n[Fe_4S_4(DmpS)_4]$ (*n*=0-4) recorded on powdered samples at room-temperature. The fitting contributions are shown as *grey lines*; the rising edge, simulated as a pseudo-Voigt line, as a *dotted* one, the pre-edge peak as a *dashed* one and the mid-edge peak as a *dashed-dotted* one. The total fit is represented by a *solid grey line*. Refer to Table S3 for a compilation of selected parameters of the fits.

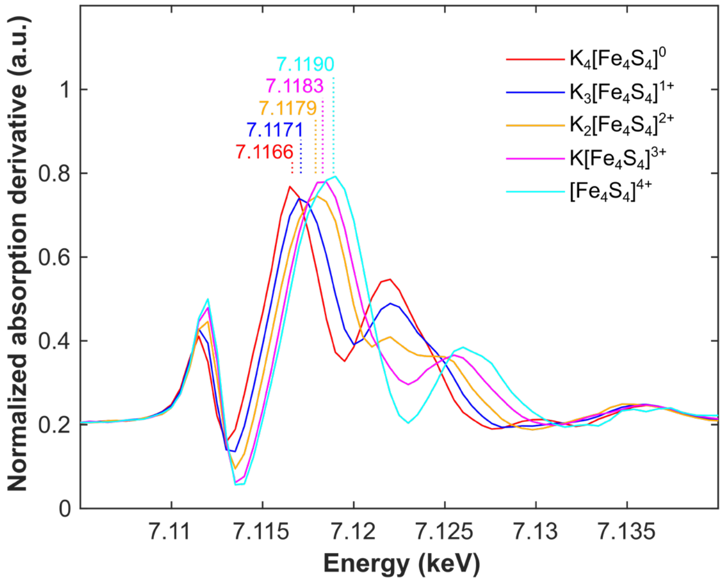

Figure S5. Normalized and 1st derivative Fe K-edge XAS spectra of $K_n[Fe_4S_4(DmpS)_4]$ (n=0-4) recorded on powdered samples at room-temperature. E_0-values (in eV) are indicated by *dotted colored lines* at their respective positions in the 1st derivative spectra.

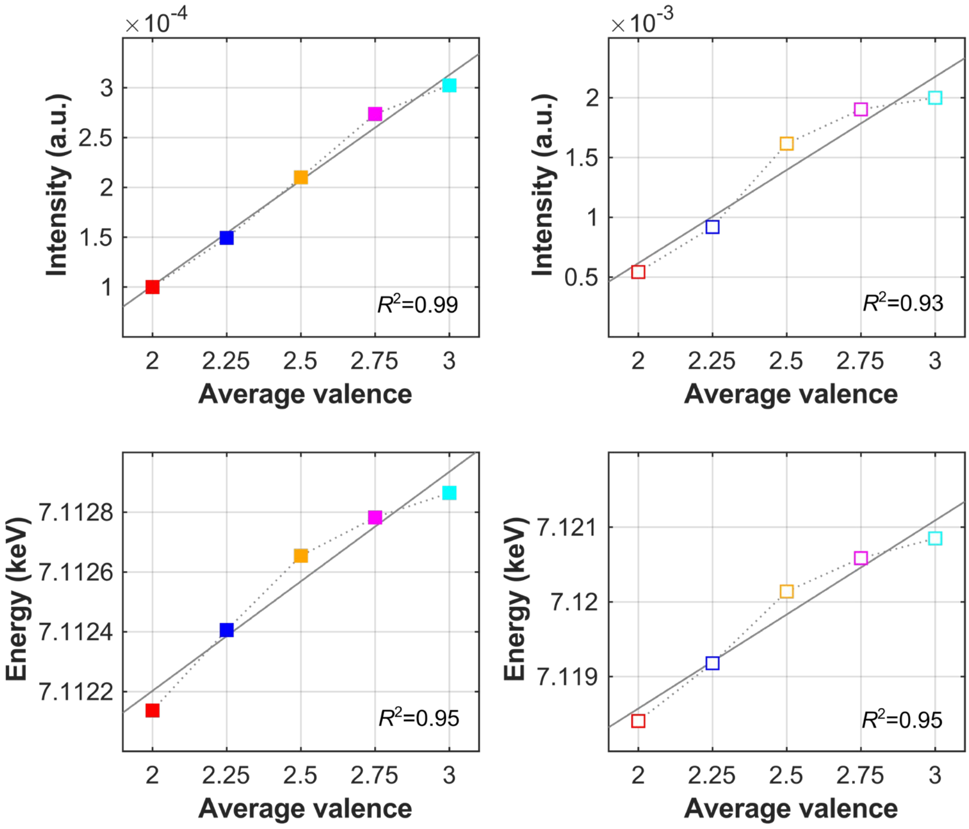

Figure S6. Variation of the absorption features (pre-edge: *solid squares*; mid-edge: *hollow squares*) in the Fe K-edge spectra with the average valence of the Fe atoms according to the model utilizing a pseudo-Voigt line to simulate the absorption edge. (*Top*) variation of the intensity (integral); (*bottom*) variation of the peak energy position. Linear fits (*grey lines*) indicate a gain of 0.18±0.02 eV per additional hole for the pre-edge peak's energy position and of 0.63±0.08 eV for the energy of the mid-edge transition. *Dotted grey lines* guide the eye.

Table S3. Summary of Fe K-edge data analysis and fitting parameters.

complex	E_0 (eV)[a]	pre-edge energy (eV)[b]	pre-edge intensity (a.u.)[b]	mid-edge energy (eV)[b]	mid-edge intensity (a.u.)[b]
$[Fe_4S_4]^{4+}$	7119.0	7112.9	$0.30\cdot10^{-3}$	7120.8	$2.00\cdot10^{-3}$
$K[Fe_4S_4]^{3+}$	7118.3	7112.8	$0.27\cdot10^{-3}$	7120.6	$1.90\cdot10^{-3}$
$K_2[Fe_4S_4]^{2+}$	7117.9	7112.7	$0.21\cdot10^{-3}$	7120.1	$1.61\cdot10^{-3}$
$K_3[Fe_4S_4]^{1+}$	7117.1	7112.4	$0.15\cdot10^{-3}$	7119.2	$0.92\cdot10^{-3}$
$K_4[Fe_4S_4]^{0}$	7116.6	7112.1	$0.10\cdot10^{-3}$	7118.4	$0.54\cdot10^{-3}$

[a] Determined from the inflection point as shown in the 1st derivative of the absorption spectra (Figure S5). [b] Determined from fitting of the absorption edge features (*vide supra*; Figure S4).

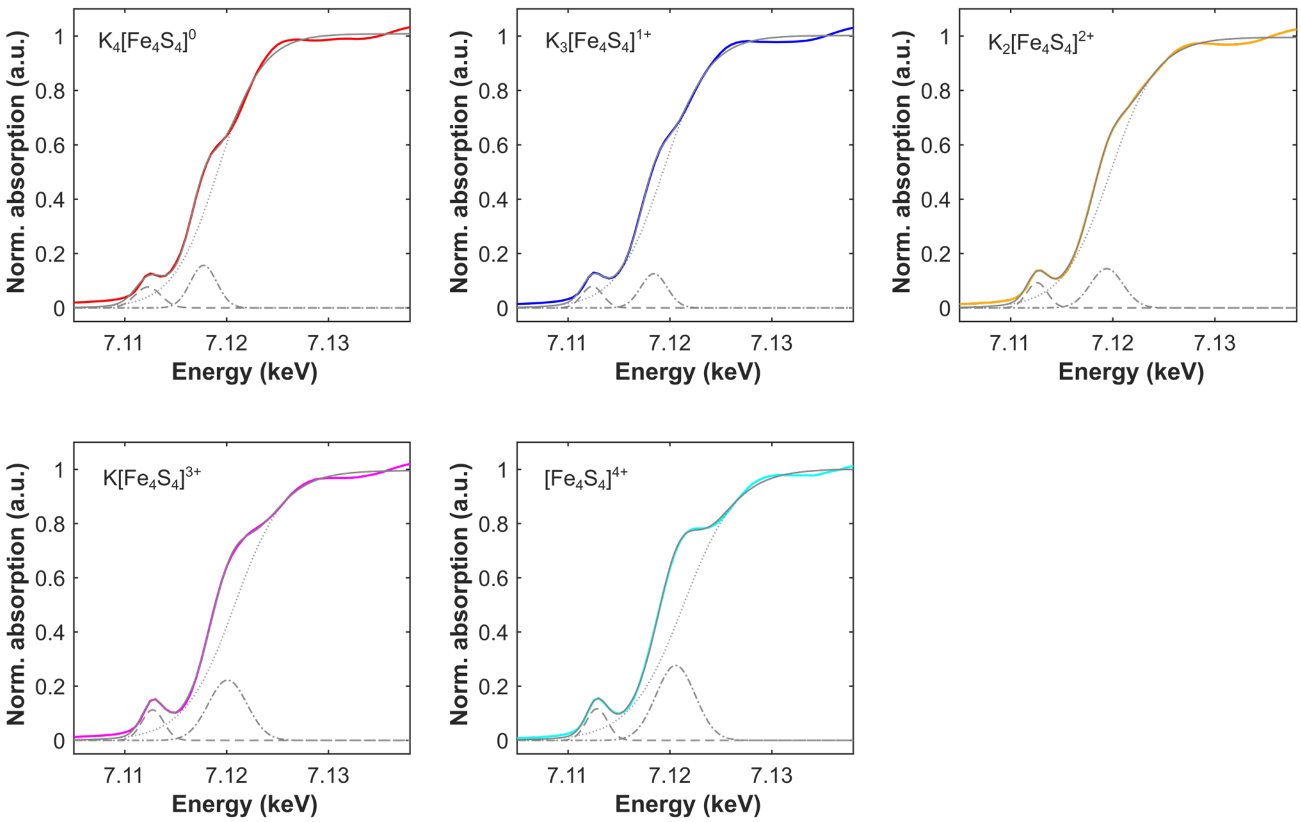

Figure S7. Normalized and fitted Fe K-edge XAS spectra of $K_n[Fe_4S_4(DmpS)_4]$ (n=0-4) recorded on powdered samples at room-temperature. The fitting contributions are shown as *grey lines*; the rising edge, simulated as a smoothed step function, as a *dotted* one, the pre-edge peak as a *dashed* one and the mid-edge peak as a *dashed-dotted* one. The total fit is represented by a *solid grey line*. Refer to Table S4 for a compilation of selected parameters of the fits.

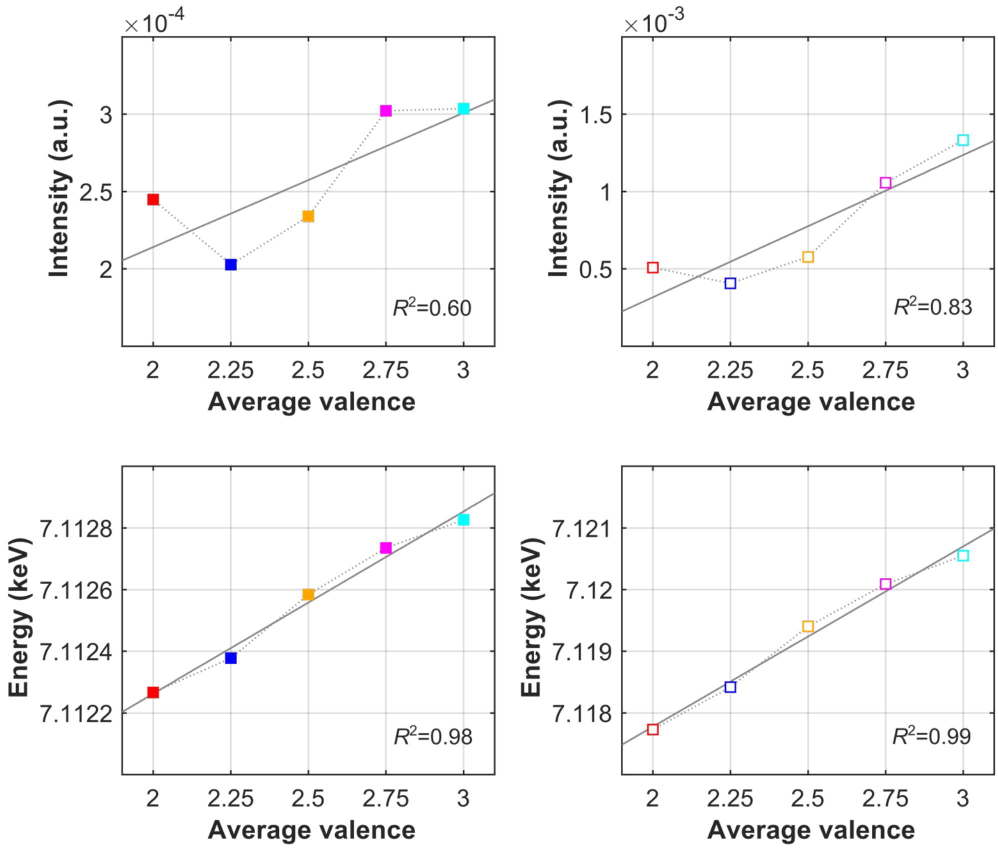

Figure S8. Variation of the absorption features (pre-edge: *solid squares*; mid-edge: *hollow squares*) in the Fe K-edge spectra with the average valence of the Fe atoms according to the model utilizing a step-function to simulate the absorption edge. (*Top*) variation of the intensity (integral); (*bottom*) variation of the peak energy position. Linear fits (*grey lines*) indicate a gain of 0.15±0.01 eV per additional hole for the pre-edge peak's energy position and of 0.73±0.05 eV for the energy of the mid-edge transition. *Dotted grey lines* guide the eye.

Table S4. Summary of Fe K-edge data analysis and fitting parameters.

complex	E_0 (eV)[a]	pre-edge energy (eV)[b]	pre-edge intensity (a.u.)[b]	mid-edge energy (eV)[b]	mid-edge intensity (a.u.)[b]
$[Fe_4S_4]^{4+}$	7121.3	7112.8	$0.30\cdot10^{-3}$	7120.6	$1.33\cdot10^{-3}$
$K[Fe_4S_4]^{3+}$	7120.8	7112.7	$0.30\cdot10^{-3}$	7120.1	$1.06\cdot10^{-3}$
$K_2[Fe_4S_4]^{2+}$	7119.8	7112.6	$0.23\cdot10^{-3}$	7119.4	$0.58\cdot10^{-3}$
$K_3[Fe_4S_4]^{1+}$	7119.3	7112.4	$0.20\cdot10^{-3}$	7118.4	$0.41\cdot10^{-3}$
$K_4[Fe_4S_4]^{0}$	7119.1	7112.3	$0.24\cdot10^{-3}$	7117.7	$0.51\cdot10^{-3}$

[a] Determined from the fitted XANES spectra, where the absorption edge was modelled by a smoothed step function (Figure S7). [b] Determined from fitting of the absorption edge features (*vide supra*; Figure S7).

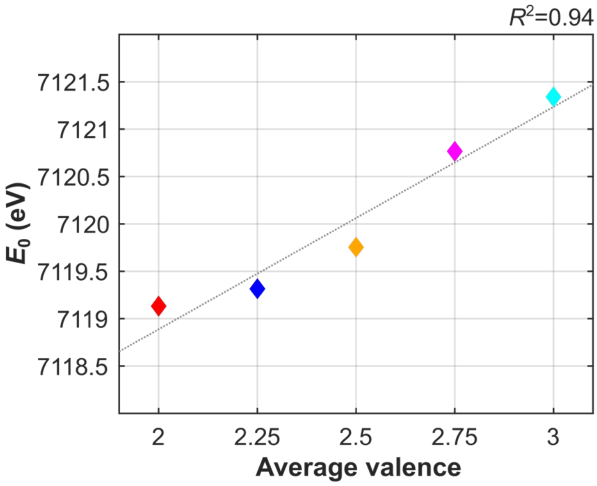

Figure S9. Variation of the mid-point of the smoothed step function modelling the absorption edge with the average valence of the Fe atoms. A linear fit (*dotted grey line*) indicates a gain of E_0 of 0.59±0.08 eV per additional hole in the Fe_4S_4 core.

Note S3. In analogy to the linear relationship derived for the XANES data from analysis of the position of the inflection point, the Fe-valence relates to E_0 derived from the position of the mid-point of the smoothed step-function (background in the deconvolution shown in Figure S7) as follows:

$$\frac{E_0 - 7114.19\text{ eV}}{2.35\text{ eV}} = \boldsymbol{V}_{\text{Fe}}$$

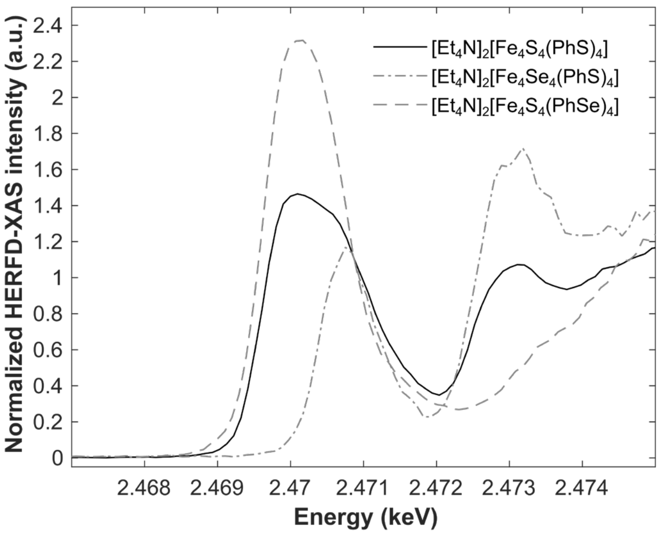

Figure S10. Normalized S K-edge HERFD-XAS spectra of powder samples of $[Et_4N]_2[Fe_4S_4(PhS)_4]$ (*solid black line*), $[Et_4N]_2[Fe_4Se_4(PhS)_4]$ (*dashed-dotted grey line*) and $[Et_4N]_2[Fe_4S_4(PhSe)_4]$ (*dashed grey line*) measured at room-temperature *in vacuo*.

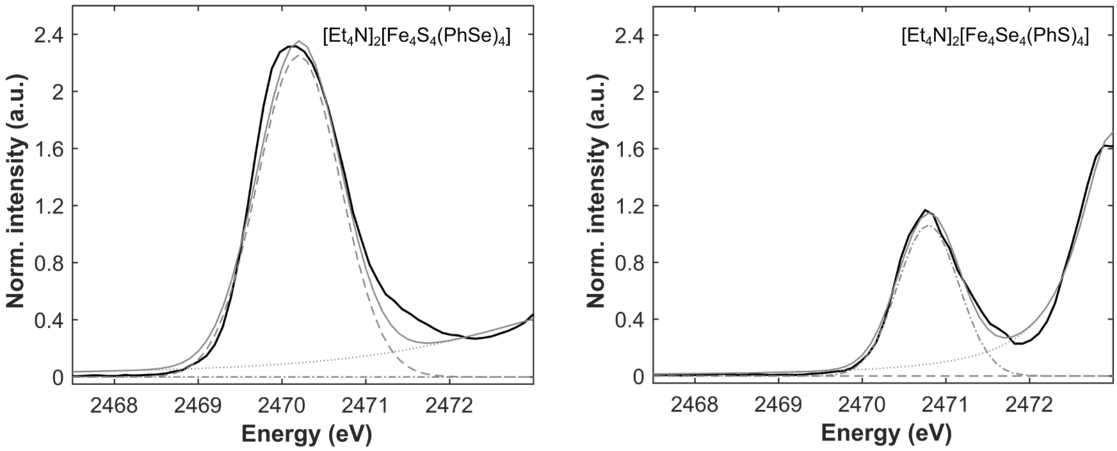

Figure S11. Fitted S K-edge HERFD-XAS spectra (*bold black lines*) of powder samples of $[Et_4N]_2[Fe_4S_4(PhSe)_4]$ (*left*) and $[Et_4N]_2[Fe_4Se_4(PhS)_4]$ (*right*) measured at room-temperature *in vacuo*. The fitting contributions are shown as *grey lines*; the rising edge as a *dotted* one, the thiolate's contribution as a *dashed-dotted* one and the sulfide's contribution as a *dashed* one. The total fit is represented by a *solid grey line*. As described above, in text, the obtained dipole strengths correspond to covalency values of 43±2% for the sulfide (literature:[9-11] 39±3-41±2%) and 36±1% for the thiolate (literature:[9-11] 35±2%), in fair agreement with values derived based on conventional XAS.

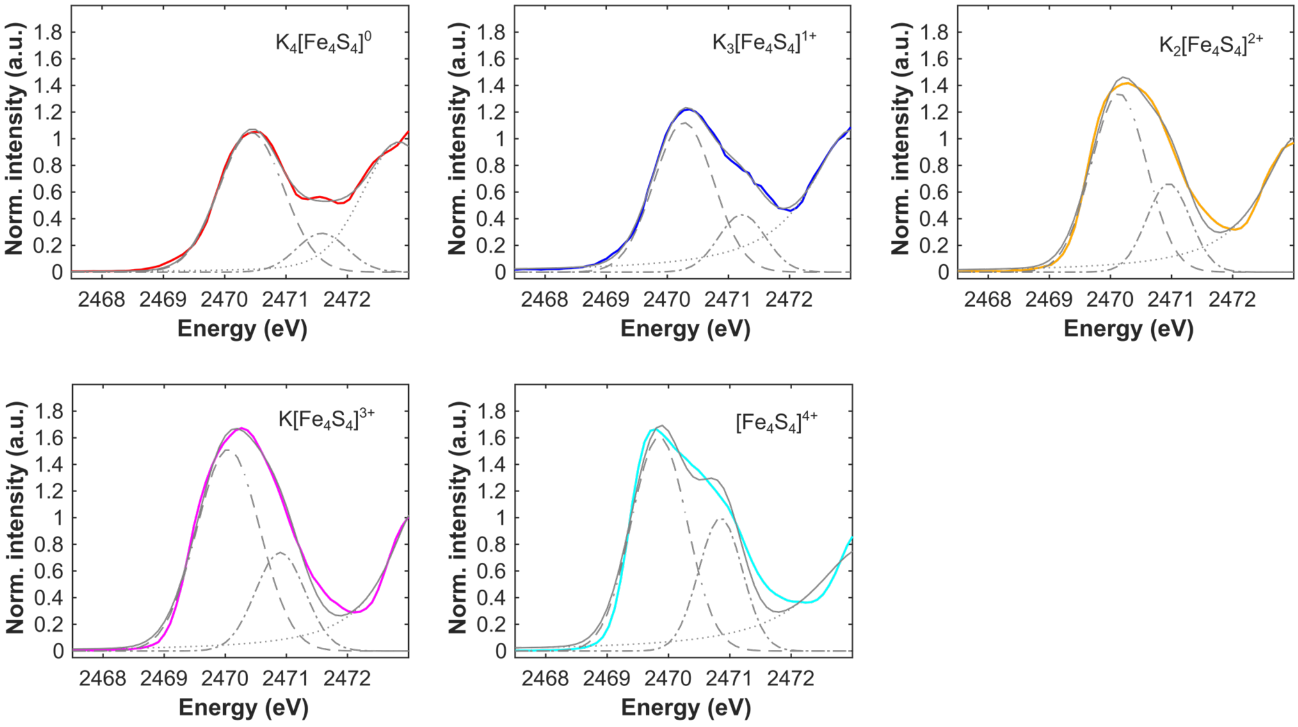

Figure S12. Normalized and fitted S K-edge HERFD-XAS spectra of $K_n[Fe_4S_4(DmpS)_4]$ (n=0-4) recorded on powdered samples at room-temperature *in vacuo*. The fitting contributions are shown as *grey lines*; the rising edge as a *dotted* one, the thiolate's contribution as a *dashed-dotted* one and the sulfide's contribution as a *dashed* one. The total fit is represented by a *solid grey line*. Refer to Table S5 for a compilation of selected parameters of the fits.

Table S5. Results of the pre-edge peak fitting routine, as shown in Figure S12.

complex	sulfide[a]				thiolate[b]			
	peak energy (eV)	intensity (a.u.)	dipole strength per bond (D_0)[c]	covalency per bond (α^2 in %)[a]	peak energy (eV)	intensity (a.u.)	dipole strength per bond (D_0)[d]	covalency per bond (α^2 in %)[b]
$[Fe_4S_4]^{4+}$	2469.9±0.1	1.83±0.14	1.22±0.09	**56±4**	2470.8±0.1	0.89±0.08	1.78±0.16	**67±6**
$K[Fe_4S_4]^{3+}$	2470.0±0.1	1.95±0.11	1.30±0.07	**60±3**	2470.8±0.1	0.75±0.03	1.50±0.06	**56±3**
$K_2[Fe_4S_4]^{2+}$	2470.1±0.1	1.52±0.09	1.01±0.06	**46±3**	2470.9±0.1	0.59±0.03	1.19±0.06	**44±3**
$K_3[Fe_4S_4]^{1+}$	2470.3±0.1	1.37±0.09	0.91±0.06	**42±2**	2471.2±0.1	0.42±0.02	0.83±0.04	**31±2**
$K_4[Fe_4S_4]^0$	2470.4±0.1	1.37±0.06	0.91±0.04	**42±1**	2471.6±0.1	0.30±0.01	0.60±0.02	**22±1**

[a] For the sulfide contribution, a transition dipole moment of 6.54 was used to calculate covalency, as determined by independent spectroscopic techniques in the literature on the infinite chain compounds $KFeS_2$ and $CsFeS_2$.[13] [b] For the thiolate contribution, a transition dipole moment of 8.05 was used to calculate covalency, as determined by independent spectroscopic techniques in the literature on the blue copper protein plastocyanine.[12-13] [c] The renormalized dipole strength of the sulfide contribution (per bond) is obtained by multiplication of the intensity by a factor of 2/3. [d] The renormalized dipole strength of the thiolate contribution (per bond) is obtained by multiplication of the intensity by a factor of 2.

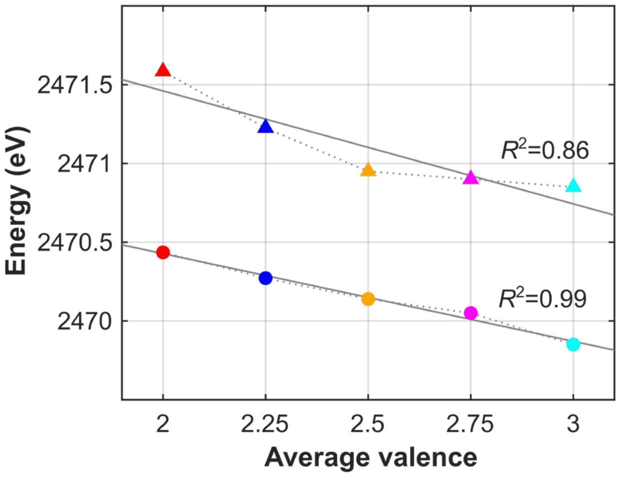

Figure S13. Variation of the pre-edge peak energy positions (*triangles*: Fe-S(thiolate) peak; *dots*: Fe-S(μ^3) peak) with the average valence of the Fe atoms for the respective oxidation states: *red*, $K_4[Fe_4S_4]^0$; *blue*, $K_3[Fe_4S_4]^{1+}$; *yellow*, $K_2[Fe_4S_4]^{2+}$; *magenta*, $K[Fe_4S_4]^{3+}$ and *cyan*, $[Fe_4S_4]^{4+}$. *Dotted lines* guide the eye and linear fits to the data are shown as *grey lines*. These linear fits indicate a loss of 0.18±0.04 eV for the thiolate peak and 0.14±0.01 eV for the sulfide peak per additional hole.

Table S6. Summary of total ligand character in the Fe 3*d* holes, ligand character per hole, and relative changes in ligand character upon redox within the FeS cubane redox series. Theoretically, a total of no.(holes) x 100% are available.

complex	total ligand character (%)[a]	number of holes[b]	ligand character per hole (%)	relative change in total ligand character on redox[c]
$[Fe_4S_4]^{4+}$	940±72	20	**47±4**	+29%
$K[Fe_4S_4]^{3+}$	944±48	19	**50±2**	+30%
$K_2[Fe_4S_4]^{2+}$	728±48	18	**40±3**	0%
$K_3[Fe_4S_4]^{1+}$	628±32	17	**37±2**	-14%
$K_4[Fe_4S_4]^{0}$	592±16	16	**37±1**	-19%

[a] Composed of 12 x the covalency of the Fe-S(μ^3) bond, and 4 x the covalency of the Fe-S(thiolate). [b] Assuming that Fe^{II} bears 4 holes, and Fe^{III} 5. [c] Given in percent (%) relative to the total covalency of all Fe-S bonds in $K_2[Fe_4S_4(DmpS)_4]$.

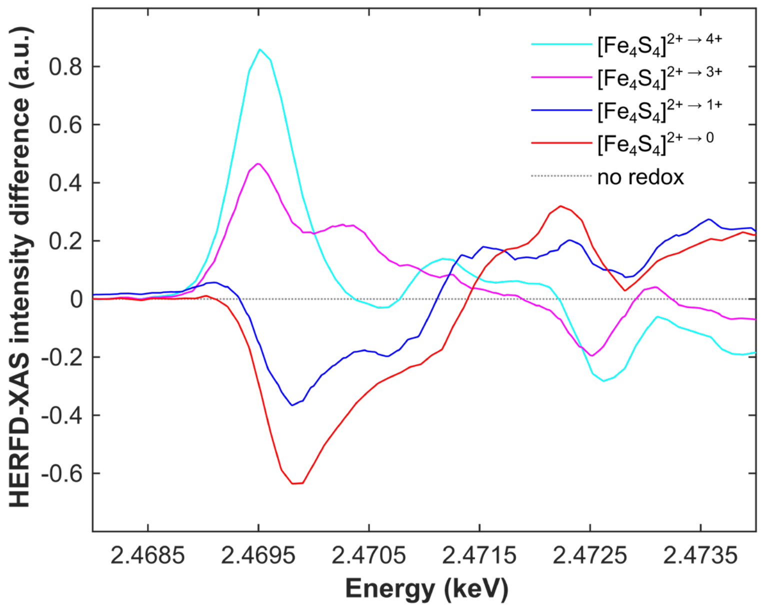

Figure S14. Normalized S K-edge HERFD-XAS difference spectra of the 1- (*magenta*/*blue*), respectively 2-electron (*cyan*/*red*) oxidized/reduced congeners of the iron-sulfur cubane with respect to the resting oxidation state spectrum of $K_2[Fe_4S_4(DmpS)_4]$. The baseline (corresponding to no redox event taking place) is represented by a *dotted grey line*.

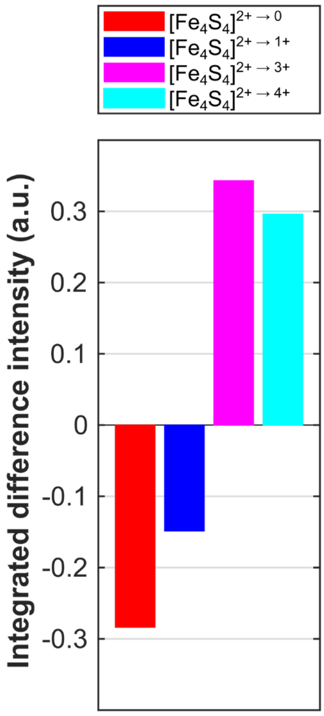

Figure S15. Net change in the intensity of the pre-edge peak upon redox transformations of the $[Fe_4S_4]^{2+}$ cubane: superreduction to $[Fe_4S_4]^0$ (*red*), ferredoxin (Fd) type reduction to $[Fe_4S_4]^{1+}$ (*blue*), high-potential iron-sulfur protein (HiPIP) type oxidation to $[Fe_4S_4]^{3+}$ (*magenta*) and superoxidation to $[Fe_4S_4]^{4+}$ (*cyan*). Note that in this way, the contribution of the rising edge to the integral (effectively the background) is not considered.

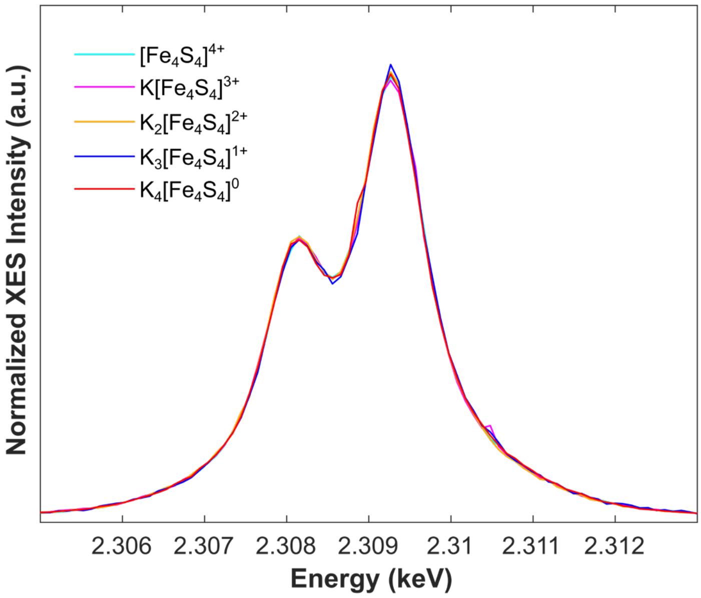

Figure S16. Normalized S K_α XES spectra of $K_n[Fe_4S_4(DmpS)_4]$ (n=0-4) measured at room-temperature on powdered samples *in vacuo*.

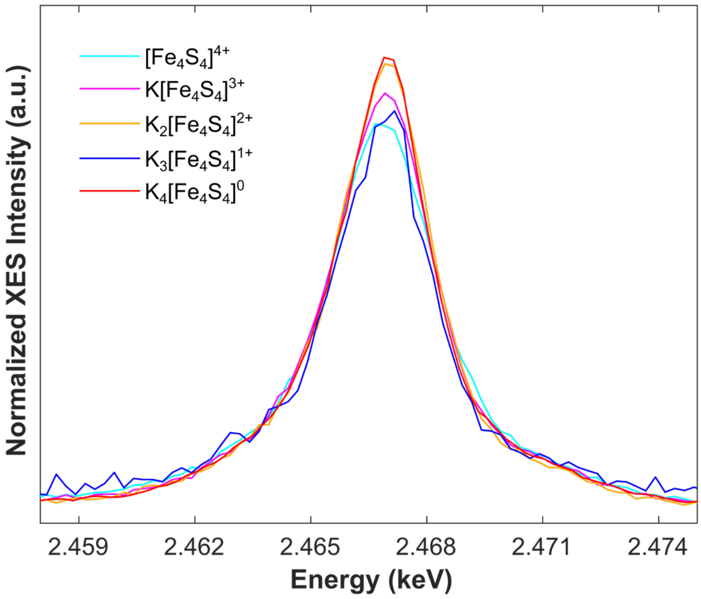

Figure S17. Normalized S K_β XES spectra of $K_n[Fe_4S_4(DmpS)_4]$ (n=0-4) measured at room-temperature on powdered samples *in vacuo*.

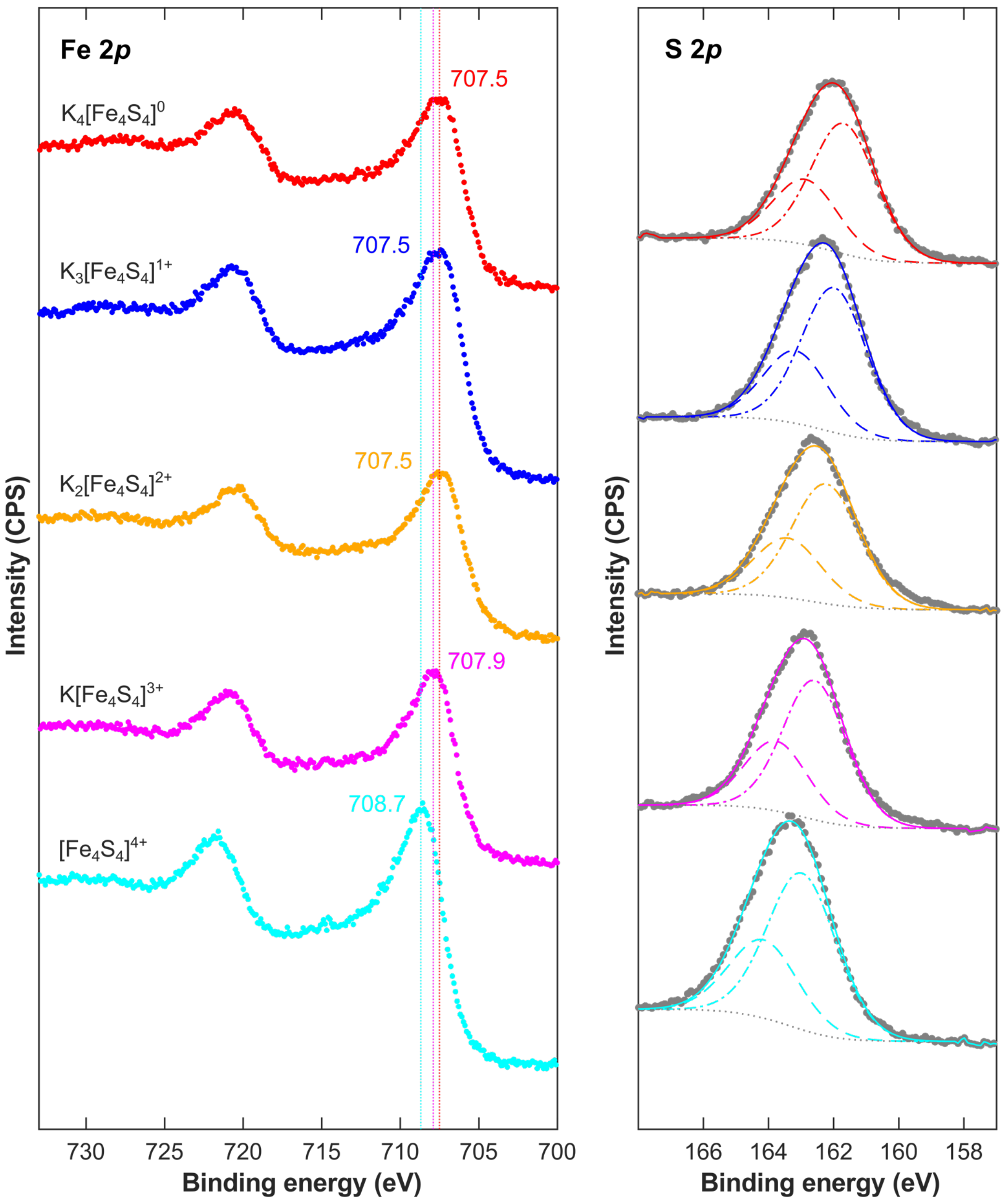

Figure S18. Core-level Fe 2*p* (*left*) and S 2*p* (*right*), XPS spectra for $K_n[Fe_4S_4(DmpS)_4]$ (*n*=0-4) referenced to the C 1s peak (at 284.8 eV) of each complex. *Coloured dotted lines* indicate the energy position of the maximum of the Fe $2p_{3/2}$ emission lines, and the corresponding values are given alongside. The *dotted grey line* is the background (type “Shirley”), while coloured *dashed* and *dashed-dotted lines* represent the fits to the S $2p_{1/2}$ and S $2p_{3/2}$ peaks, respectively. All data is shown as *dots* (*coloured* for the Fe 2*p* data and *grey* for the S 2*p* data), while the total fits are *solid coloured lines*. A summary of the fitted parameters is given in Table S7.

Table S7. Summary of the energetic fitting parameters for the core-level Fe 2$p_{3/2}$ and S 2p XPS spectra.

complex	Fe 2$p_{3/2}$ E (eV)	S 2p E (eV)[a]
$[Fe_4S_4]^{4+}$	708.7	163.4
$K[Fe_4S_4]^{3+}$	707.9	163.0
$K_2[Fe_4S_4]^{2+}$	707.5	162.6
$K_3[Fe_4S_4]^{1+}$	707.5	162.4
$K_4[Fe_4S_4]^0$	707.5	162.1

[a] Refers to the energy position of the maximum of the total S 2p fit.

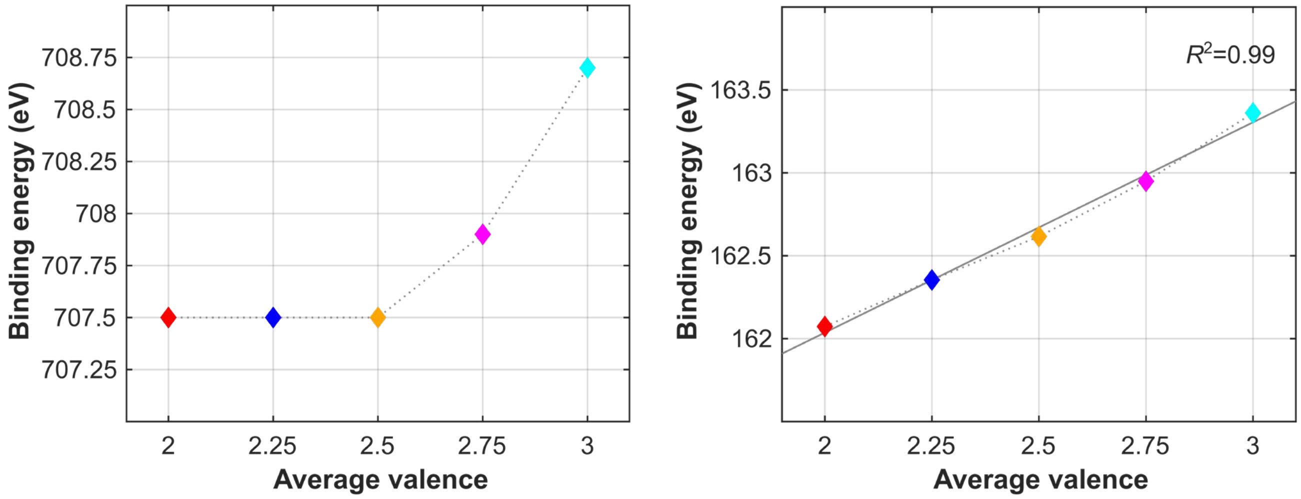

Figure S19. (*Left*) variation of the Fe 2$p_{3/2}$ peak energy (coloured *diamonds*) with the average valence of the Fe atoms. (*Right*) variation of the S 2p XPS Binding energy with the average valence of the Fe atoms. Data are shown as *coloured diamonds*, and a *grey line* indicates a linear fit to the data. Each 1-electron oxidation causes a shift of 0.32±0.02 eV to the maximum of the transition.

References

1. Grunwald, L.; Clémancey, M.; Klose, D.; Dubois, L.; Gambarelli, S.; Jeschke, G.; Wörle, M.; Blondin, G.; Mougel, V., A complete biomimetic iron-sulfur cubane redox series. *Proceedings of the National Academy of Sciences* **2022,** *119* (31), e2122677119.

2. Grunwald, L.; Inoue, M.; Carril, P. C.; Wörle, M.; Mougel, V., Gated electron transfers at synthetic iron-sulfur cubanes. *Chem* **2024,** *10* (1), 365-387.

3. Abbott, D.; Grunwald, L.; Singh-Morgan, A.; Walker, A.; Xu, Y.-Z., Investigating the Electrochemical Reduction of Nitrate to Ammonia on Single-Atom Catalysts via Operando X-ray Absorption Spectroscopy. In *Investigating the Electrochemical Reduction of Nitrate to Ammonia on Single-Atom Catalysts via Operando X-ray Absorption Spectroscopy*, Facility, E. S. R., Ed. European Synchrotron Radiation Facility, 2025.

4. Ravel, B.; Newville, M., ATHENA, ARTEMIS, HEPHAESTUS: data analysis for X-ray absorption spectroscopy using IFEFFIT. *Journal of Synchrotron Radiation* **2005,** *12* (4), 537-541.

5. Abbott, D.; Grunwald, L.; Singh-Morgan, A., Combined X-ray Absorption and X-ray Emission Spectroscopy Study of Synthetic [Fe_4S_4] Cubane Complexes at the S K-edge. In *Combined X-ray Absorption and X-ray Emission Spectroscopy Study of Synthetic [Fe_4S_4] Cubane Complexes at the S K-edge*, Facility, E. S. R., Ed. European Synchrotron Radiation Facility, 2026.

6. Rovezzi, M.; Harris, A.; Detlefs, B.; Bohdan, T.; Svyazhin, A.; Santambrogio, A.; Degler, D.; Baran, R.; Reynier, B.; Noguera Crespo, P.; Heyman, C.; Van Der Kleij, H.-P.; Van Vaerenbergh, P.; Marion, P.; Vitoux, H.; Lapras, C.; Verbeni, R.; Kocsis, M. M.; Manceau, A.; Glatzel, P., TEXS: in-vacuum tender X-ray emission spectrometer with 11 Johansson crystal analyzers. *Journal of Synchrotron Radiation* **2020,** *27* (3), 813-826.

7. Solé, V. A.; Papillon, E.; Cotte, M.; Walter, P.; Susini, J., A multiplatform code for the analysis of energy-dispersive X-ray fluorescence spectra. *Spectrochimica Acta Part B: Atomic Spectroscopy* **2007,** *62* (1), 63-68.

8. Averill, B. A.; Herskovitz, T.; Holm, R. H.; Ibers, J. A., Synthetic analogs of the active sites of iron-sulfur proteins. II. Synthesis and structure of the tetra[mercapto-μ^3-sulfido-iron] clusters, $[Fe_4S_4(SR)_4]^{2-}$. *Journal of the American Chemical Society* **1973,** *95* (11), 3523-3534.

9. Glaser, T.; Rose, K.; Shadle, S. E.; Hedman, B.; Hodgson, K. O.; Solomon, E. I., S K-edge X-ray Absorption Studies of Tetranuclear Iron−Sulfur Clusters: μ-Sulfide Bonding and Its Contribution to Electron Delocalization. *Journal of the American Chemical Society* **2001,** *123* (3), 442-454.

10. Dey, A.; Glaser, T.; Couture, M. M. J.; Eltis, L. D.; Holm, R. H.; Hedman, B.; Hodgson, K. O.; Solomon, E. I., Ligand K-Edge X-ray Absorption Spectroscopy of $[Fe_4S_4]^{1+,2+,3+}$ Clusters: Changes in Bonding and Electronic Relaxation upon Redox. *Journal of the American Chemical Society* **2004,** *126* (26), 8320-8328.

11. Glaser, T.; Bertini, I.; Moura, J. J. G.; Hedman, B.; Hodgson, K. O.; Solomon, E. I., Protein Effects on the Electronic Structure of the $[Fe_4S_4]^{2+}$ Cluster in Ferredoxin and HiPIP. *Journal of the American Chemical Society* **2001,** *123* (20), 4859-4860.

12. Shadle, S. E.; Hedman, B.; Hodgson, K. O.; Solomon, E. I., Ligand K-edge x-ray absorption spectroscopic studies: metal-ligand covalency in a series of transition metal tetrachlorides. *Journal of the American Chemical Society* **1995,** *117* (8), 2259-2272.

13. Rose, K.; Shadle, S. E.; Glaser, T.; de Vries, S.; Cherepanov, A.; Canters, G. W.; Hedman, B.; Hodgson, K. O.; Solomon, E. I., Investigation of the Electronic Structure of 2Fe−2S Model Complexes and the Rieske Protein Using Ligand K-Edge X-ray Absorption Spectroscopy. *Journal of the American Chemical Society* **1999,** *121* (11), 2353-2363.

14. Solomon, E. I.; Hedman, B.; Hodgson, K. O.; Dey, A.; Szilagyi, R. K., Ligand K-edge X-ray absorption spectroscopy: covalency of ligand–metal bonds. *Coordination Chemistry Reviews* **2005,** *249* (1), 97-129.

15. Fairley, N.; Fernandez, V.; Richard‐Plouet, M.; Guillot-Deudon, C.; Walton, J.; Smith, E.; Flahaut, D.; Greiner, M.; Biesinger, M.; Tougaard, S.; Morgan, D.; Baltrusaitis, J., Systematic and collaborative approach to problem solving using X-ray photoelectron spectroscopy. *Applied Surface Science Advances* **2021,** *5*, 100112.
